# A MIF-p38-GSDMD inflammatory loop in keratinocytes underlies UVB-induced cutaneous lupus

**DOI:** 10.1038/s41419-026-08443-4

**Published:** 2026-02-02

**Authors:** Chipeng Guo, Siweier Luo, Jigang Luo, Siyao Lu, Xiaomei You, Junlin Cao, Yufei Du, Haoran Lv, Hanzhi Liang, Le Wang, Liangchun Wang, Tao Liu, Yiming Zhou

**Affiliations:** 1 Department of Dermatology, Sun Yat-sen Memorial Hospital, Sun Yat-sen University, Guangzhou, China; 2 Basic and Translational Medical Research Center, Sun Yat-sen Memorial Hospital, Sun Yat-sen University, Guangzhou, China; 3 Medical College of Acu-Moxi and Rehabilitation, Guangzhou University of Chinese Medicine, Guangzhou, China; 4 Department of Nephrology, The First Affiliated Hospital, Sun Yat-sen University, Guangzhou, China

**Keywords:** Autoimmune diseases, Autoimmunity

## Abstract

Ultraviolet B (UVB) is a well-recognized trigger of cutaneous lupus erythematosus (CLE), yet its molecular basis remains largely undefined. Here, using single-cell transcriptomics and a lupus-prone mouse model, we identify keratinocyte-derived macrophage migration inhibitory factor (MIF) as a key amplifier of cutaneous inflammation through a self-sustaining feedback loop. Single-cell RNA sequencing reveals elevated MIF expression specifically within pathogenic, interferon-high keratinocyte subclusters associated with CLE, which is further validated across major CLE subtypes in clinical skin samples. In vitro, UVB irradiation dose-dependently induces the release of MIF from keratinocytes, which in turn promotes inflammatory signaling and matrix remodeling in both keratinocytes and fibroblasts. Mechanistically, we demonstrate that UVB irradiation activates the ribotoxic stress response (RSR), leading to the p38-C/EBPβ-mediated transcriptional upregulation of NLRP3 and GSDMD cleavage in keratinocytes. The ensuing GSDMD-dependent pyroptosis facilitates the release of MIF, primarily through GSDMD pores rather than vesicular secretion, which in turn amplifies the p38-C/EBPβ signaling pathway. Therapeutic disruption of this loop either by gene silencing via AAVs or pharmacological inhibition via microneedles, markedly attenuates epidermal hyperplasia and cytokine imbalance in lupus-prone mice. These findings uncover a previously unrecognized MIF-p38-GSDMD inflammatory loop contributes to the UVB-induced cutaneous lupus, offering both mechanistic insights and translational opportunities for CLE.

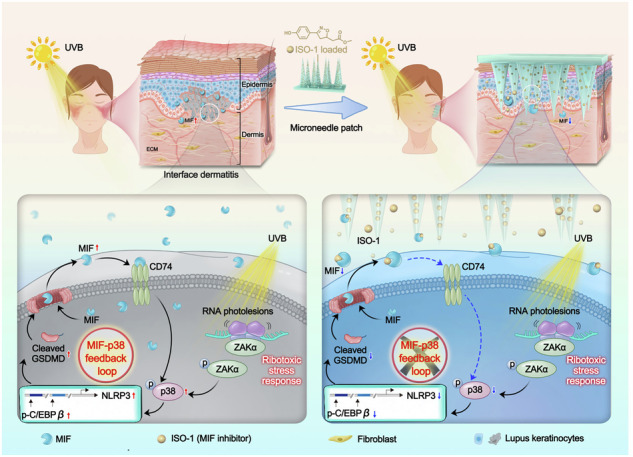

## Introduction

Systemic lupus erythematosus (SLE) is a devastating autoimmune disease characterized by systemic inflammation and multi-organ damage, representing a significant global health challenge with profound socioeconomic implications [1–3]. Among its diverse clinical manifestations, cutaneous lupus erythematosus (CLE), marked by chronic, disfiguring skin lesions, is one of the most common and frequently the earliest clinical sign [2, 4, 5]. CLE pathology is defined by aberrant tissue remodeling and inflammation [2], driven by the dysregulated expression of matrix metalloproteinases (e.g., MMP2 and MMP9) [6] and pro-inflammatory cytokines (e.g., TNF-α and IL1β) [7]. While previous research has largely focused on infiltrating immune cells in CLE pathogenesis, a paradigm shift is underway, recognizing the keratinocyte as an active instigator of cutaneous inflammation [8, 9]. This is supported by recent single-cell transcriptomic studies revealing that lupus keratinocytes exhibit aberrant, pro-inflammatory gene expression signatures even prior to lesion formation [10, 11]. The emerging recognition of keratinocytes as primary drivers of pathology underscores the potential limitation of current treatments that predominantly target systemic immunity, and the urgent need for novel mechanistic insights and targeted therapies.

Ultraviolet B (UVB) is a well-recognized environmental trigger for CLE, clinically termed as photosensitivity [2]. Photosensitivity is not only a diagnostic hallmark of lupus [2] but also a critical factor necessitating rigorous photoprotection in patients [12]. However, the molecular mechanisms linking UVB irradiation to CLE pathogenesis remain unclear [4, 13, 14]. With a wavelength range of 280–320 nm, UVB predominantly targets the epidermal layer [14], where keratinocytes, the principal cellular constituents, are hypothesized to orchestrate its pathological effects [8]. Emerging evidence suggests that keratinocytes in lupus patients exhibit intrinsic hyperresponsiveness to UVB, leading to sustained inflammation and tissue damage [15, 16]. However, the precise molecular pathways within keratinocytes that translate the physical stress of UVB into a sustained, self-amplifying inflammatory response remain unclear [4, 13], which limits the development of specific therapies targeting the initial cutaneous disease drivers.

Macrophage migration inhibitory factor (MIF), a pleiotropic cytokine with potent pro-inflammatory properties, has emerged as a pivotal player in SLE pathogenesis [17, 18]. Genetic polymorphisms in *MIF* are strongly associated with SLE susceptibility [19–22], and elevated circulating MIF levels correlate with disease severity [23]. Within the skin, MIF derived from keratinocytes has been implicated in driving tissue remodeling and inflammatory responses [24, 25]. Previous studies have suggested that UVB could modulate MIF in keratinocytes [26, 27], where MIF has been shown to regulate tissue inflammation and matrix remodeling [28, 29]. However, a comprehensive understanding of its cell-type-specific expression pattern in CLE lesions, its regulation by UVB, and its precise role in the pathogenic feedback loop has been lacking.

In this study, leveraging single-cell transcriptomics, we first identified keratinocyte-derived MIF as the dominantly upregulated cytokine within specific, expanded keratinocyte subpopulations in CLE patient skins, providing a strong rationale for its further investigation. We demonstrate that UVB triggers the release of MIF from lupus keratinocytes, driving tissue remodeling and inflammation via autocrine and paracrine signaling. Mechanistically, we demonstrate that UVB irradiation engages stress signaling leading to the upregulation of NLRP3 expression and GSDMD cleavage through the p38-C/EBPβ signaling pathway in keratinocytes. Notably, we identify a switch from the canonical NLRP1-mediated pyroptosis in normal keratinocytes to a p38-C/EBPβ-NLRP3-mediated pathway in the lupus context. The ensuing GSDMD-dependent pyroptosis facilitates the release of MIF, which in turn amplifies the p38-C/EBPβ signaling pathway, thereby establishing a self-sustaining inflammatory loop. Therapeutic intervention using intradermal delivery of a *Mif*-shRNA by adeno-associated virus (AAV) or a MIF inhibitor by innovative, dissolvable microneedle patches effectively disrupted this feedback loop, attenuating UVB-induced skin lesions in lupus-prone mice. Collectively, our findings elucidate a previously unrecognized MIF-p38-GSDMD mechanism in CLE pathogenesis and establish MIF as a promising therapeutic target.

## Results

### scRNA-seq analysis showed lupus-specific keratinocyte subclusters with elevated expression of macrophage migration inhibitory factor (MIF)

**Fig. 1:**
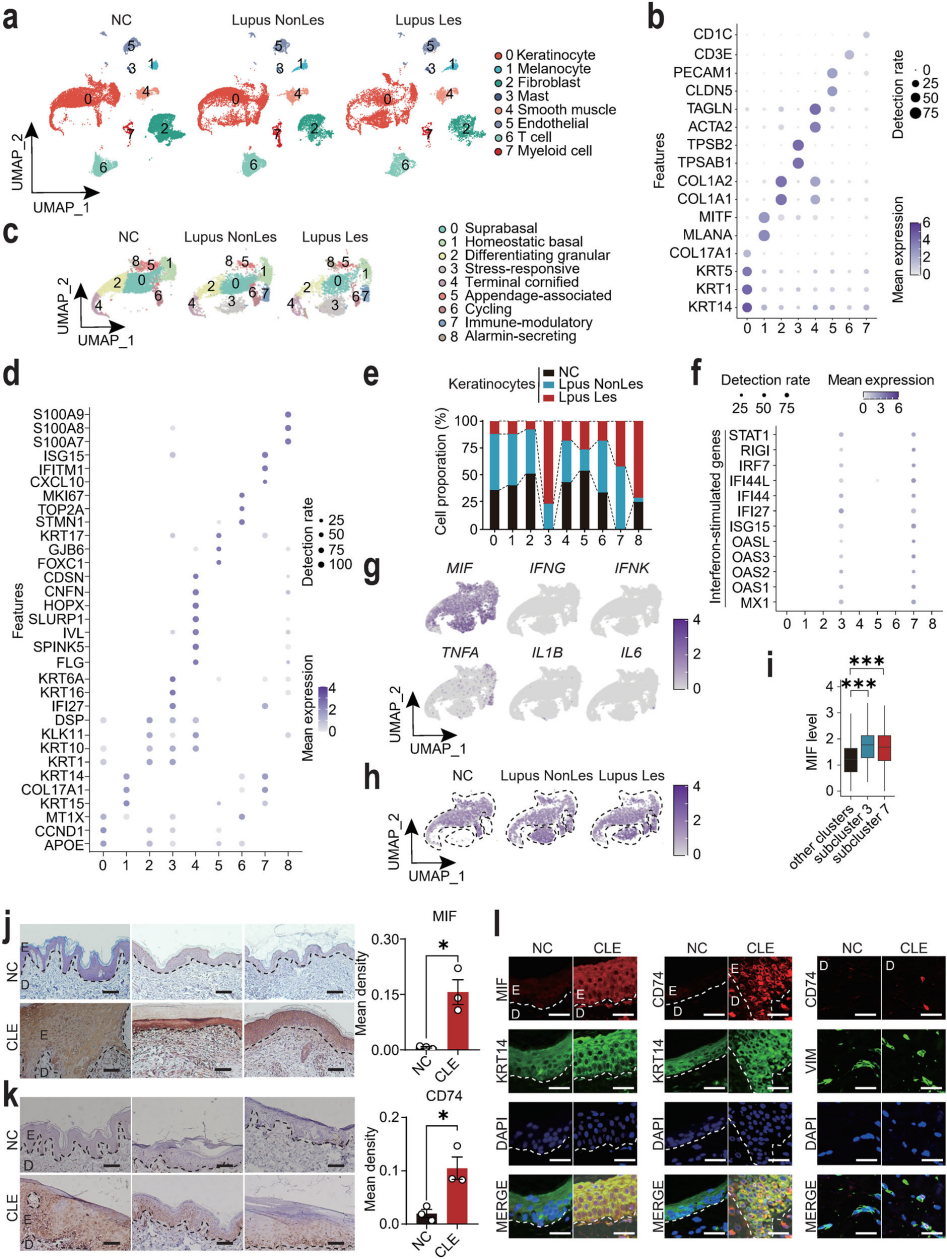
Single-cell transcriptomic profiling identifies lupus-specific keratinocyte subclusters with elevated macrophage migration inhibitory factor (MIF). **a** UMAP projection of single-cell clusters annotated for major cutaneous cell types in normal controls (NC, *N* = 14), lupus non-lesional (Lupus NonLes, *N* = 7), and lupus lesional skin (Lupus Les, *N* = 7). **b** Dot plot of differentially expressed genes across clusters (color scale: mean expression; dot size: detection rate). **c** Unsupervised subclustering of keratinocytes identifies nine transcriptionally distinct subpopulations. **d** Dot plot of subcluster-specific marker genes (color scale: mean expression; dot size: detection rate). **e** Bar plot showing proportions of keratinocyte subclusters across NC, Lupus NonLes, and Lupus Les groups. **f** Dot plot of interferon-stimulated genes (ISGs) expression in keratinocyte subclusters. **g** Cytokine expression profiles of lupus-associated cytokines across subclusters. **h** Spatial mapping of MIF expression in keratinocyte subclusters across NC, Lupus NonLes, and Lupus Les groups. **i** Box-and-whisker plots comparing MIF expression levels between lupus-specific subclusters 3 and 7 versus combined remaining subclusters (0, 1, 2, 4, 5, 6, 8). Immunohistochemical staining of MIF (**j**) and CD74 (**k**) in normal skin (NC) and cutaneous lupus erythematosus tissues (CLE) (*N* = 3 each). E: epidermis; D: dermis. **l** Immunofluorescence co-localization of MIF/CD74 with keratinocyte marker KRT14 and fibroblast marker VIM in normal skins (NC) and CLE tissues. E: epidermis; D: dermis. Data are mean ± SEM. Scale bars = 100 μm. ^*^P < 0.05, ^***^P < 0.001.

To investigate the cellular and molecular mechanisms underlying CLE, we analyzed scRNA-seq data (GEO: GSE186476) from 14 normal control samples and 7 paired samples of lupus lesional and non-lesional skin tissues. Through unsupervised clustering and uniform manifold approximation and projection (UMAP) dimensionality reduction, we systematically mapped the cutaneous cellular architecture into eight principal lineages (Fig. 1a, b). Given the established role of keratinocytes as primary UVB sensors orchestrating photosensitivity responses in CLE pathogenesis [9, 30], we performed high-resolution subclustering, revealing nine transcriptionally distinct keratinocyte subclusters (Fig. 1c, d). Comparative analysis showed significant expansion of stress-responsive (subcluster 3) and immune-modulatory (subcluster 7) keratinocytes in both lesional and non-lesional lupus skin compared to healthy controls (Fig. 1e). Notably, these two subclusters exhibited distinct interferon-stimulated gene (ISG) signatures, marked by elevated expression of *MX1*, *OAS1*, *OAS2*, *OAS3*, *OASL*, *ISG15*, *IFI27*, *IFI44*, *IFI44L*, *IRF7*, *RIGI*, and *STAT1*, that were absent in other keratinocyte subclusters (Fig. 1f), which align with characteristic lupus pathophysiology [31, 32]. Given the pathogenic relevance of these expanded, interferon-high subclusters in lupus, we sought to identify their key effector molecules. Analysis of their cytokine secretory profiles revealed macrophage migration inhibitory factor (*MIF*) as the most dominantly upregulated effector molecule in contrast to the minimal expression of classical pro-inflammatory cytokines (*IFNG*, *IFNK*, *TNFA*, *IL1B*, *IL6*) observed in keratinocytes (Fig. 1g). This selective upregulation was solidified by the significant increase of *MIF* mRNA specifically in the pathogenic subclusters 3 and 7 from both lesional and non-lesional lupus skins compared to all other subclusters combined (Fig. 1h, i). This pattern, marked by prominent MIF upregulation prior to overt lesion formation and specifically within the disease-relevant subpopulations, suggests its intrinsic link to the interferon response and its potential role as an early pathogenic mediator in lupus progression.

Immunohistochemistry (IHC) validated a marked increase in MIF and its receptor CD74 expression in the epidermis of CLE patients compared to normal controls (Fig. 1j, k). Given that the scRNA-seq dataset was derived from a cohort containing subacute cutaneous lupus erythematosus (SCLE) and discoid lupus erythematosus (DLE, a form of chronic CLE) but not acute cutaneous lupus erythematosus (ACLE) samples, we sought to determine whether MIF upregulation is a general feature across CLE subtypes. We therefore performed additional IHC staining of MIF on skin tissues from patients with ACLE, SCLE, and chronic cutaneous lupus erythematosus (CCLE), along with normal controls. Quantitative analysis demonstrated significantly elevated MIF expression in the epidermis across all three CLE subtypes compared to normal skin (Fig. S1), confirming that MIF upregulation is a consistent feature in CLE regardless of subtype classification. Co-staining experiments with the keratinocyte marker keratin 14 (KRT14) and fibroblast marker vimentin (VIM) demonstrated higher expression of MIF and CD74 in lupus keratinocytes, with CD74 also detectable in lupus fibroblasts (Fig. 1l). These results were further supported by qRT-PCR analysis, which confirmed the upregulation of MIF signaling components (*MIF* and *CD74*), tissue remodeling factors (*COL1A1*, *MMP2*, and *MMP9*), and inflammatory mediators (*IL1B*, *TNFA*, *IFNG*, and *MX1*) in CLE lesional skin, with modest increases observed in dermatomyositis (DM) and Lichen sclerosus et atrophicus (Fig. S2a). Furthermore, correlation analysis of gene expression in normal and CLE lesional skins revealed that *MIF* levels are significantly positively correlated with those of *TNFA*, *IL1B*, *MMP2*, *MMP9*, *COL1A1*, *MX1*, and *IFNK*, but not *IL6* (Fig. S2b), underscoring the close relationship between MIF and key inflammatory and tissue-remodeling pathways in CLE. Collectively, these findings validate our transcriptomic discovery, identifying the expanded, interferon-high keratinocyte subpopulations as a major source of MIF in lupus-affected skins. The specific and pronounced dysregulation of MIF within these pathogenic subclusters, coupled with its consistent protein-level upregulation across CLE subtypes, underscores its role beyond general homeostasis and positions it as a compelling candidate driver of pathological remodeling and inflammation in CLE.

### UVB irradiation induces the release of keratinocyte-derived MIF that amplifies the inflammatory and remodeling responses

**Fig. 2:**
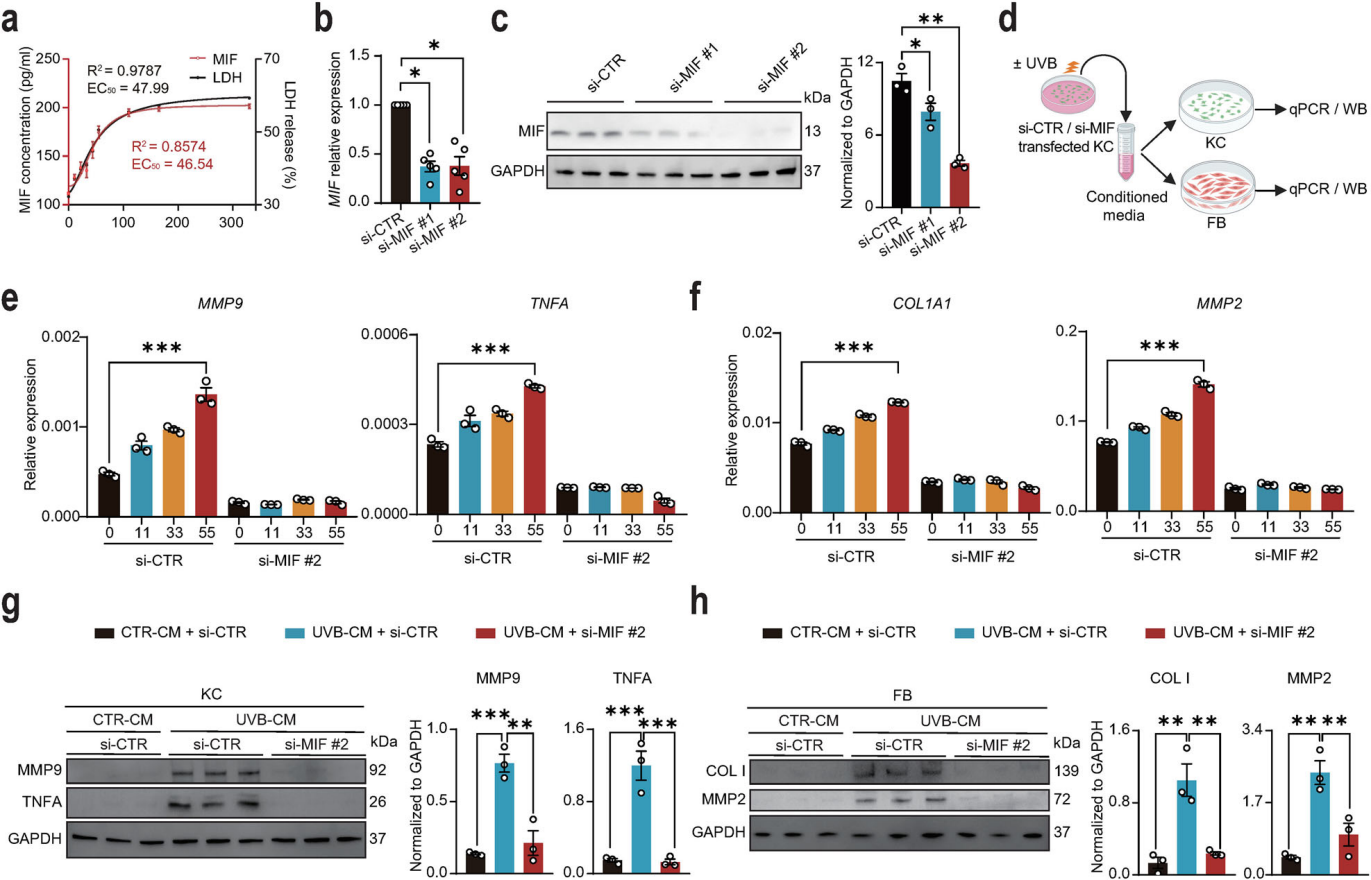
UVB-induced MIF release from keratinocytes exacerbates skin inflammation and tissue remodeling. **a** ELISA quantification of MIF in supernatants 12 h post-UVB exposure (11, 22, 33, 44, 55, 110, 165, and 330 mJ/cm^2^). Dose-response fitted by four-parameter logistic model (EC₅₀ = 46.54 mJ/cm², *R*² = 0.8574) (*N* = 6 each). LDH release was measured in parallel (EC₅₀ = 47.99 mJ/cm², *R*² = 0.9787) (*N* = 6 each). **b** mRNA expression levels of MIF in HaCaT cells 24 h post-transfection with control-siRNA (si-CTR) or MIF-siRNAs (si-MIF #1 and #2) (*N* = 5 each). **c** Protein levels and quantification of MIF in HaCaT cells 48 h post-transfection with si-CTR or MIF-siRNAs (si-MIF #1 and #2) (*N* = 3 each). **d** Schematic of the experimental design. Conditioned media (CM) were collected from UVB-irradiated keratinocytes with or without MIF knockdown and then applied to fresh keratinocytes (KC) and fibroblasts (FB) to assess the paracrine effects of MIF. **e** mRNA levels of MMP9 and TNFA in keratinocytes treated with conditioned media (CM) from HaCaT cells that were pre-exposed to the indicated doses of UVB (11, 33, and 55 mJ/cm²), with or without MIF knockdown (*N* = 3 each). **f** mRNA levels of COL1A1 and MMP2 in fibroblasts treated with CM from HaCaT cells that were pre-exposed to UVB, with or without MIF knockdown (*N* = 3 each). Protein levels and quantification of MMP9 and TNF-α in keratinocytes (**g**), and COL I and MMP2 in fibroblasts (**h**). Cells were treated with conditioned media (CM) from untreated (CTR-CM) or UVB-exposed HaCaT cells (UVB-CM), with or without MIF knockdown (*N* = 3 each). Data are mean ± SEM. ^*^P < 0.05, ^**^P < 0.01, ^***^P < 0.001.

While our analysis of patient lesions identified MIF as a key upregulated factor in pathogenic keratinocytes (Fig. 1), its regulatory relationship with classical pro-inflammatory cytokines under chronic disease conditions remained unclear. We hypothesized that MIF, as a pre-formed damage-associated molecular pattern, might exert its primary effects upon release following acute cellular stress, rather than through constitutive co-expression with downstream cytokines [33–36]. Given that UVB irradiation is a well-established key environmental trigger of CLE [4, 13], we next sought to mechanistically dissect how this clinically relevant stimulus drives MIF release and amplifies inflammation. Although previous studies have suggested that UVB irradiation may regulate MIF expression [26, 27], we found that MIF expression in keratinocytes remained unchanged following UVB irradiation, regardless of exposure intensity or duration (Fig. S3a, b). Interestingly, we discovered that UVB irradiation dose-dependently triggered MIF release into the culture medium, with a four-parameter logistic model confirming a robust dose-response relationship (EC₅₀ = 46.54 mJ/cm², *R*² = 0.8574) (Fig. 2a). The magnitude of MIF release was highly correlated with that of lactate dehydrogenase (LDH), a classic indicator of plasma membrane permeabilization (EC₅₀ = 47.99 mJ/cm², *R*² = 0.9787), suggesting that MIF efflux is a passive consequence of membrane disruption (Fig. 2a). To determine the functional consequences of UVB-induced MIF release, we first knocked down MIF expression in keratinocytes using siRNA, which significantly reduced both MIF mRNA and protein levels (Fig. 2b, c). Conditioned media from untreated and UVB-irradiated si-CTR or si-MIF keratinocytes were applied to naïve keratinocytes and fibroblasts (Fig. 2d). We analyzed a panel of markers encompassing key aspects of CLE pathology: MMP2 and MMP9 for tissue remodeling, TNFα for inflammation, and COL1 for fibrosis [6, 7]. Conditioned media from UVB-irradiated keratinocytes dose-dependently upregulated the mRNA expression levels of *MMP9* and *TNFA* in keratinocytes and *COL1A1* and *MMP2* in fibroblasts (Fig. 2e, f). Notably, genetic knockdown of MIF significantly attenuated this upregulation (Fig. 2e, f). These findings were further corroborated at the protein level (Fig. 2g, h). The attenuation of these effects by si-MIF conditioned media confirms the specific role of keratinocyte-derived MIF in this process. Complementary pharmacological inhibition of MIF with ISO-1 yielded a similar attenuation of these pro-inflammatory and matrix-remodeling responses (Fig. S4a, b). Collectively, these findings demonstrate that UVB may exacerbate skin pathology by promoting the release of pre-synthesized MIF from keratinocytes, which in turn amplifies inflammatory and matrix-remodeling responses. This mechanism explains the dominant upregulation of MIF in patient keratinocytes and uncovers its context-dependent role that is unmasked upon pathogenic stress.

### Ribotoxic stress response (RSR) induced by UVB irradiation triggers a MIF-p38-GSDMD inflammatory loop in lupus keratinocytes

**Fig. 3:**
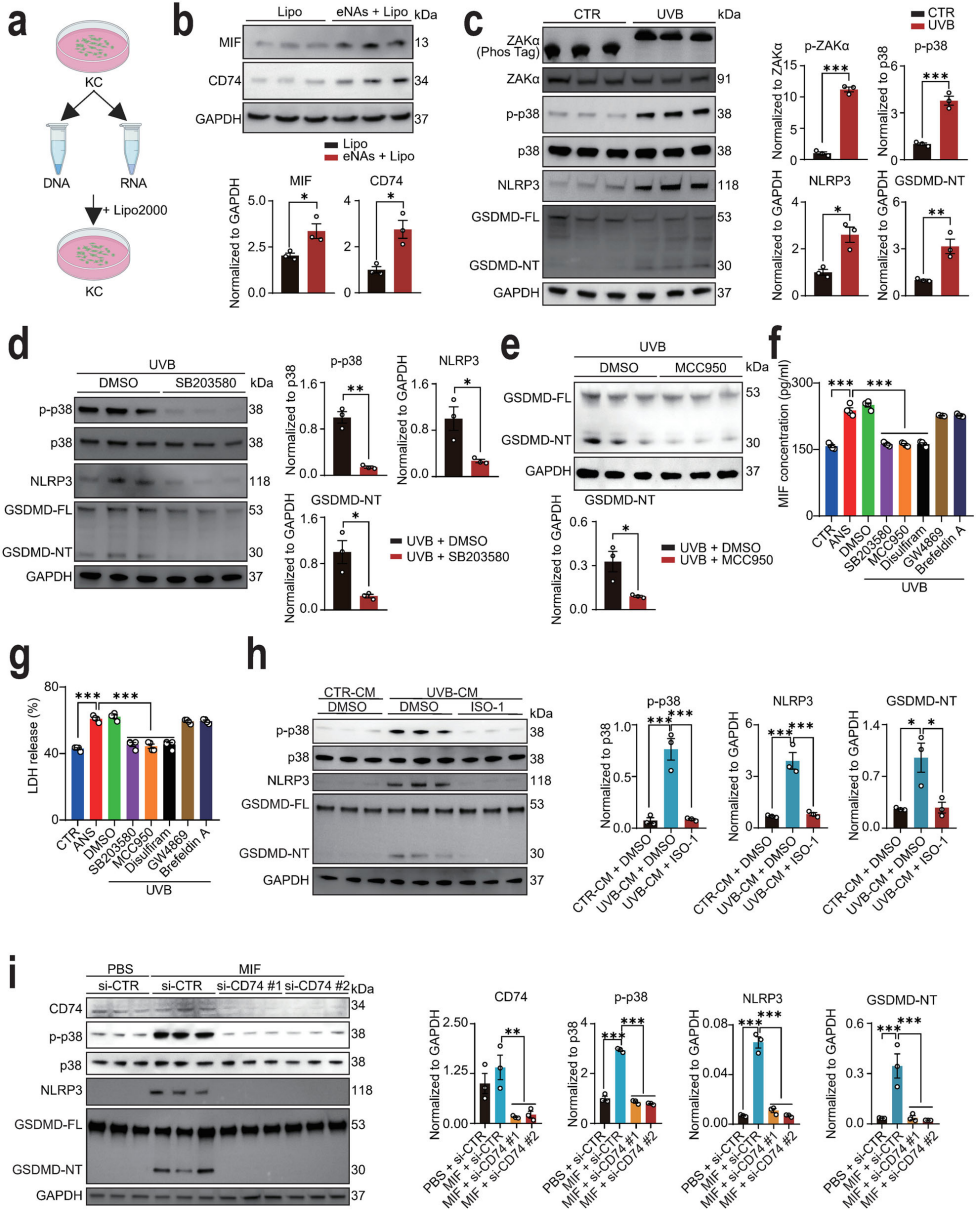
UVB irradiation triggers a MIF-p38-GSDMD inflammatory loop in lupus keratinocytes. **a** Schematic of experimental design to generate lupus-prone keratinocytes via transfection of HaCaT cells with endogenous nucleic acids (eNAs). **b** Protein levels and quantification of MIF and CD74 in eNAs-transfected HaCaT cells (*N* = 3 each). **c** Protein levels and quantification of p-ZAKα, ZAKα, p-p38, p38, NLRP3, and GSDMD (full-length, FL; N-terminal fragment, NT) in eNAs-transfected HaCaT cells with or without UVB exposure (55 mJ/cm²) (*N* = 3 each). **d** Protein levels and quantification of p-p38, p38, NLRP3, GSDMD-FL, and GSDMD-NT in UVB-exposed eNAs-transfected HaCaT cells pre-treated with DMSO or p38 inhibitor SB203580 (*N* = 3 each). **e** Protein levels and quantification of GSDMD-FL and GSDMD-NT in UVB-exposed eNAs-transfected HaCaT cells pre-treated with DMSO or NLRP3 inhibitor MCC950 for 2 h (*N* = 3 each). **f** MIF secretion in supernatants of eNAs-transfected HaCaT cells quantified by ELISA under basal conditions (CTR), following ZAKα activation by anisomycin (ANS), and post-UVB exposure with DMSO, SB203580 (p38 inhibitor), MCC950 (NLRP3 inhibitor), disulfiram (DSF, GSDMD pore inhibitor), GW4869 (exosome inhibitor), or brefeldin A (Golgi inhibitor) treatment (*N* = 4 each). **g** LDH release in supernatants from the same experimental conditions as in **f** (N = 4 each). **h** Protein levels and quantification of p-p38, p38, NLRP3, and GSDMD in eNAs-transfected HaCaT cells treated with conditioned media from untreated (CTR-CM) or UVB-exposed HaCaT cells (UVB-CM), with or without ISO-1 (*N* = 3 each). **i** Protein levels and quantification of CD74, p-p38, p38, NLRP3, and GSDMD in eNAs-transfected HaCaT cells transfected with a control siRNA (si-CTR) or two CD74-targeting siRNAs (si-CD74 #1 and #2) followed by PBS or MIF treatment (*N* = 3 each). Data are mean ± SEM. ^*^P < 0.05, ^**^P < 0.01, ^***^P < 0.001.

Recent studies have identified UVB irradiation as a trigger for the ribotoxic stress response (RSR) in normal keratinocytes, leading to the activation of the ZAKα-p38 signaling pathway and subsequent GSDMD-mediated pyroptosis [37]. The formation of GSDMD pores during pyroptosis facilitates cellular lysis and the release of inflammatory cytokines [37–39]. To investigate UVB-induced RSR in the context of lupus pathogenesis, we established a disease-relevant model by transfecting HaCaT cells with endogenous nucleic acids (eNAs) (Fig. 3a), a method previously used to mimic the cytosolic self-nucleic acid accumulation characteristic of lupus keratinocytes [40, 41]. This approach recapitulates the pre-activated state of lupus keratinocytes, which are chronically exposed to eNAs due to impaired clearance and enhanced apoptosis, thereby creating a cellular context hyperresponsive to UVB. To establish the relevance of this model, we confirmed that eNAs-transfected cells recapitulate key molecular features of lupus keratinocytes. At the protein level, we observed a significant upregulation of MIF and its receptor CD74 (Fig. 3b). Furthermore, transcriptional profiling revealed a broad interferon signature (*ISG15*, *IFI44*, *RIGI*, *MX1*, *OAS1*, *OASL*, *IFI44L*, and *IFI27*), and elevated markers of pathogenic keratinocyte subpopulations (*CXCL10*, *IFITM1*, *S100A8*, and *S100A9*) (Fig. S5). This multifaceted profile closely resembles the transcriptional signature of lupus keratinocytes (Fig. 1f), thereby providing a validated system for studying lupus-specific pathways.

In this lupus-primed cellular context, UVB irradiation triggered robust activation of RSR components, including phosphorylated ZAKα (p-ZAKα), phosphorylated p38 (p-p38), and GSDMD cleavage (GSDMD-NT) (Fig. 3c). Pharmacological inhibition of p38 with SB203580 attenuated UVB-induced p-p38 and GSDMD-NT expression (Fig. 3d), confirming the critical role of p38 in this cascade. Given that UV irradiation has been reported to activate different gasdermins depending on the cellular context and stimulus characteristics [42], we employed the specific RSR activator anisomycin (ANS) to isolate the RSR pathway. Our data demonstrated that ANS treatment didn’t induce significant activation of GSDME (Fig. S6), underscoring the pathway specificity of the RSR-GSDMD axis in mediating pyroptosis in our lupus keratinocyte model.

Although NLRP1 is well-established as the primary mediator of UVB-induced pyroptosis in normal keratinocytes [37, 43, 44], we found that UVB irradiation didn’t alter the cleavage pattern of the NLRP1 in our lupus keratinocytes (Fig. S7). Instead, lupus keratinocytes exhibited pronounced UVB-induced NLRP3 upregulation (Fig. 3c), which was suppressed by p38 inhibition (Fig. 3d). Given prior evidence implicating MIF in NLRP3 inflammasome activation [33, 34, 45], we aimed to determine whether NLRP3 acts as the key effector of UVB-induced pyroptosis in lupus keratinocytes. To test this hypothesis, we treated UVB-irradiated cells with the NLRP3-specific inhibitor MCC950 and observed a significant reduction in GSDMD-NT levels (Fig. 3e). Furthermore, genetic knockdown of NLRP3 also attenuated GSDMD cleavage (Fig. S8), suggesting that NLRP3 is a critical pyroptotic mediator in this context, a mechanism absent in normal keratinocytes under comparable conditions [37, 44]. In parallel, UVB and ANS stimulation significantly enhanced MIF secretion, which was attenuated by p38 and NLRP3 inhibition (Fig. 3f). To specifically address the pathway of MIF release, we employed pharmacological inhibitors targeting key secretory mechanisms. Pretreatment with disulfiram (DSF), a specific inhibitor of GSDMD pore formation, markedly attenuated UVB-induced MIF release (Fig. 3f). This inhibition correlated strongly with reduced LDH release (Fig. 3g), confirming suppression of plasma membrane permeabilization. In contrast, inhibitors of vesicular secretion pathways (GW4869 for exosome inhibition and brefeldin A for inhibition of Golgi-mediated secretion) showed no significant effect on MIF release (Fig. 3f, g), ruling out major involvement of vesicle-dependent mechanisms. Together with the observed correlation between MIF and LDH release (Fig. 2a), these data provide consistent biochemical evidence that MIF release is primarily mediated through GSDMD pores during pyroptosis, independent of vesicular trafficking.

Given that p38 serves as a key signaling hub in keratinocytes responding to external stimuli [46, 47], we next investigated the impact of secreted MIF on lupus keratinocytes. Conditioned media from UVB-irradiated keratinocytes enhanced p38 phosphorylation in lupus keratinocytes, an effect reversed by MIF inhibitor ISO-1 (Fig. 3h). Consistent with the central role of p38 in driving the pyroptotic cascade, the upregulation of NLRP3 and cleavage of GSDMD induced by UVB-conditioned media were also abolished by ISO-1 (Fig. 3h). Genetic knockdown of CD74 similarly diminished p-p38 levels and suppressed NLRP3 and GSDMD-NT (Fig. 3i), confirming that MIF activates p38 via CD74 in an autocrine loop. Together, these findings describe a self-amplifying feedback loop in UVB-irradiated lupus keratinocytes. UVB exposure triggers the RSR that activates p38 pathway, leading to increased NLRP3 expression and GSDMD-dependent pyroptosis. This causes MIF to be released, which then binds to its receptor CD74 and further activates p38, NLRP3, and GSDMD cleavage in these cells. This feedback loop represents a previously unrecognized mechanistic link between UVB exposure and the pathogenesis of CLE.

### p38 regulates NLRP3 transcription through site-specific promoter binding via C/EBPβ

**Fig. 4:**
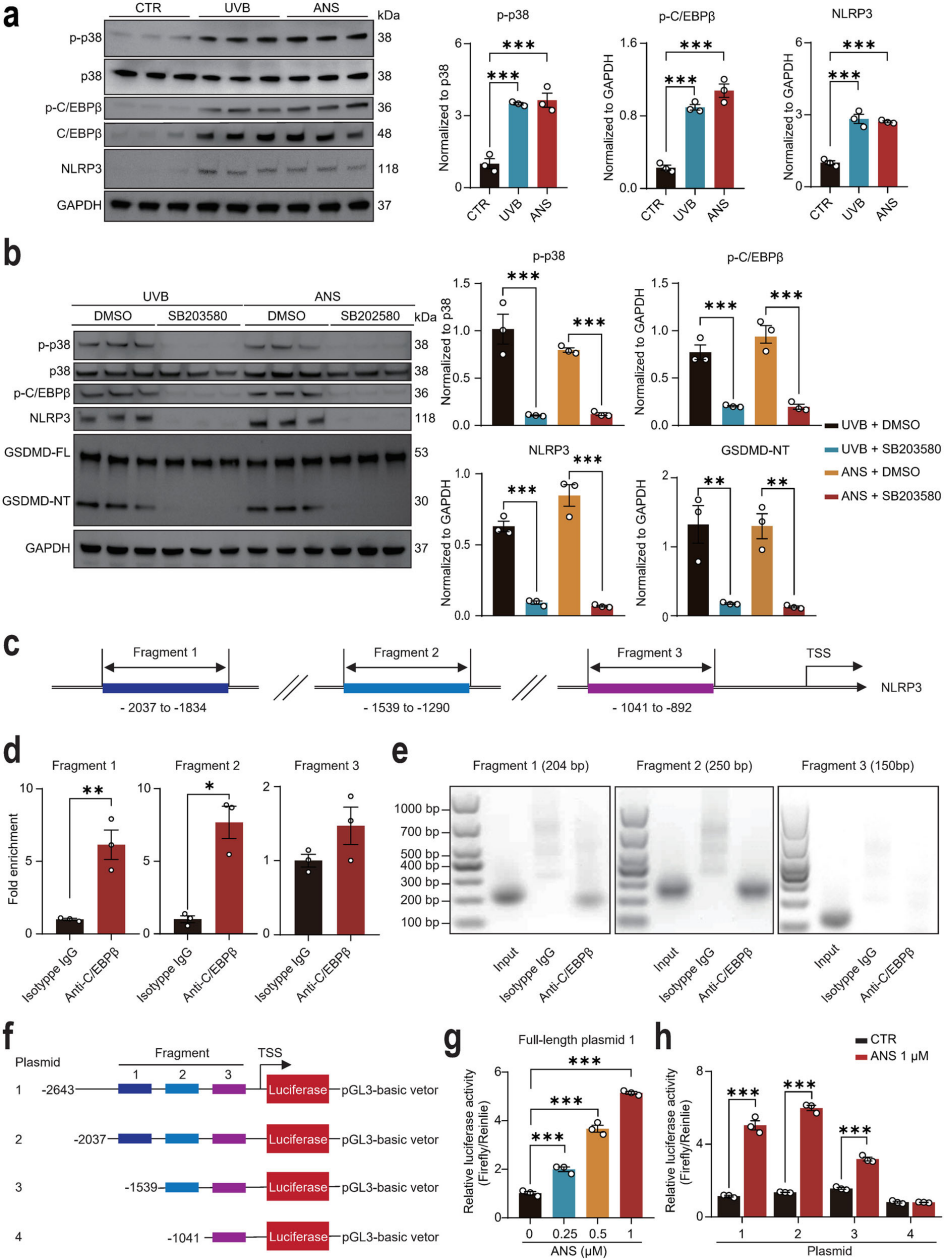
C/EBPβ directly binds the NLRP3 promoter to drive transcription upon p38 activation. **a** Protein levels and quantification of p-p38, p38, p-C/EBPβ, C/EBPβ, and NLRP3 in untreated (CTR) HaCaT cells and cells treated with UVB (55 mJ/cm²) or ZAKα activator anisomycin (ANS) (*N* = 3 each). **b** Protein levels and quantification of p-p38, p38, p-C/EBPβ, NLRP3, and GSDMD in HaCaT cells pretreated with the p38 inhibitor SB203580 followed by UVB or ANS treatment (*N* = 3 each). **c** Schematic of three human NLRP3 promoter fragments (Fragment 1: –2037 to –1834 bp; Fragment 2: –1539 to –1290 bp; Fragment 3: –1041 to –892 bp) relative to the transcription start site (TSS). **d** ChIP-qPCR quantification of C/EBPβ occupancy at NLRP3 promoter sites after ANS treatment (*N* = 3 each). **e** Gel electrophoresis of ChIP-PCR products validating C/EBPβ binding specificity. **f** Luciferase assay using four NLRP3 promoter fragments cloned into pGL3-basic luciferase vectors. **g** Dose-dependent luciferase activation by ANS (0.25, 0.5, and 1 μM) using full length NLRP3 promoter fragment (Construct 1) (*N* = 3 each). **h** Luciferase activity by ANS (1 μM) using four different promoter fragments (*N* = 3 each). Data are mean ± SEM. ^*^P < 0.05, ^**^P < 0.01, ^***^P < 0.001.

Prior studies established that p38/MAPK signaling enhances the transcriptional activity of C/EBPβ, a master regulator of inflammatory responses, including NLRP3 expression, in human keratinocytes [48–50]. Consistent with this paradigm, we found that both UVB irradiation and the ZAKα activator ANS robustly upregulated the protein levels of p-p38, total C/EBPβ, and its phosphorylated form (p-C/EBPβ), which was associated with a concomitant increase in NLRP3 expression in keratinocytes (Fig. 4a). Critically, pharmacological inhibition of p38 by SB203580 abolished the UVB- or ANS-induced upregulation of p-p38, p-C/EBPβ, NLRP3, and GSDMD cleaved, confirming that p38 activation is the upstream of these pathways (Fig. 4b). The coordinated upregulation of these proteins suggests a dual regulatory mechanism whereby p38 not only upregulates the total protein expression of C/EBPβ but also directly phosphorylates it to enhance its transcriptional activity, which is consistent with a previous study demonstrating that p38 signaling positively regulates both the total protein and phosphorylation levels of C/EBPβ in HaCaT cells [49].

To determine whether C/EBPβ directly regulates NLRP3 transcription upon p38 activation, we performed chromatin immunoprecipitation (ChIP) assays in HaCaT cells. ChIP-PCR analysis and electrophoretic gel separation of ChIP-PCR products revealed significant ANS-induced enrichment of C/EBPβ at two discrete regions of the NLRP3 promoter: Fragment 1 (−2037 to −1834 bp) and Fragment 2 (−1539 to −1290 bp) (Fig. 4c–e). However, Fragment 3 (−1041 to −892 bp) showed no detectable enrichment (Fig. 4e), indicating the absence of direct C/EBPβ binding within this promoter segment.

Next, we evaluated the functional impact of this binding through dual-luciferase reporter assays. We generated a series of NLRP3 promoter fragments (spanning −2643, −2037, −1539, or −1041 bp upstream of the transcription start site (TSS)) cloned into the pGL3-basic vector (Fig. 4f). ANS dose-dependently enhanced luciferase activity in cells transfected with the full-length NLRP3 promoter construct (−2643 bp) (Fig. 4g). Constructs containing C/EBPβ-bound regions (−2643, −2037, and −1539 bp relative to TSS) showed significant ANS-responsive activity, while the −1041 bp construct (lacking binding sites) was unresponsive **(**Fig. 4h**)**. Collectively, these data demonstrate that p38 regulates NLRP3 transcription through site-specific promoter binding via its downstream transcriptional factor C/EBPβ.

### Intradermal injection of Mif-shRNA AAV alleviated UVB-induced skin lesions of MRL/lpr mice

To investigate the in vivo effects of UVB exposure on lupus keratinocytes, we utilized a UVB-induced skin lesion model in lupus-prone MRL/lpr mice (Fig. S9a). Following UVB exposure, MRL/lpr mice developed pronounced skin lesions characterized by erythema and crusting (Fig. S9b, c), accompanied by epidermal hyperplasia and ulceration (Fig. S9b, c). IF staining revealed elevated MIF expression in keratinocytes of UVB-exposed mice (Fig. S9d), along with increased levels of COL I, MMP9, MMP2, and TNFA in skin lesions (Fig. S9e). Interestingly, studies have shown that UVB irradiation does not induce pyroptosis in keratinocytes of C57BL/6 wild-type or ZAKα−/− mice, since mouse NLRP1 is insensitive to ZAKα/p38 activation due to the absence of a critical linker region in rodent NLRP1 compared to human orthologs [37, 51], however, UVB irradiation did induce exacerbated histopathological changes in MRL/lpr lupus-prone mice. Consistent with our in vitro findings, UVB irradiation significantly upregulated the levels of MIF, p-p38, NLRP3, and GSDMD-NT in skin lesions (Fig. S9f), which indicated that UVB-induced skin lesions in lupus mice are driven by the MIF-p38-GSDMD-mediated inflammatory loop.

**Fig. 5:**
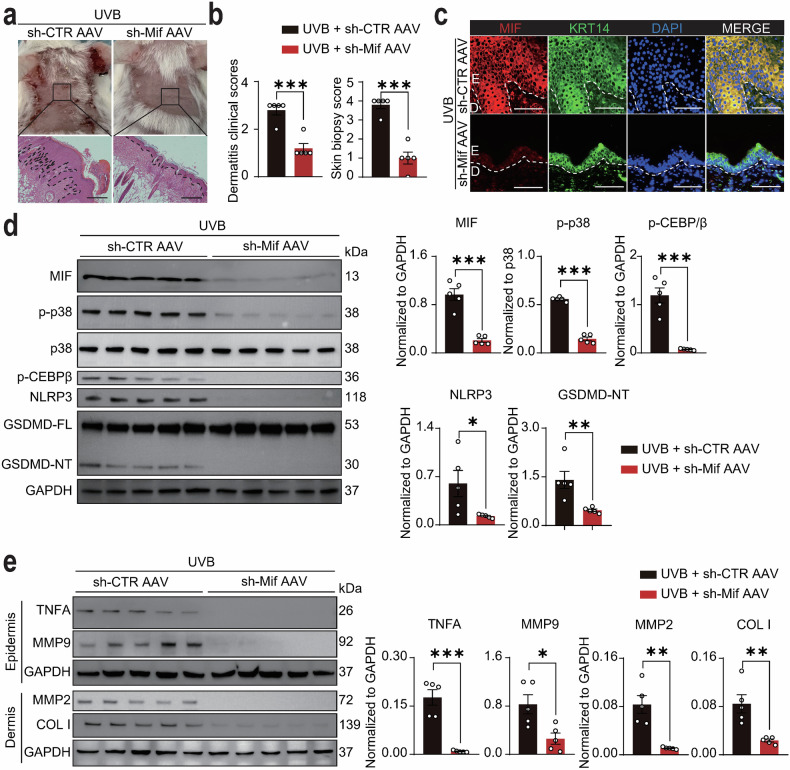
Intradermal delivery of Mif-shRNA AAV attenuates UVB-induced skin lesions in MRL/lpr mice. **a** Representative macroscopic and H&E-stained images of UVB-exposed skin in MRL/lpr mice after intradermal injection of control-shRNA AAV (sh-CTR AAV) or Mif-shRNA AAV (sh-Mif AAV) (*N* = 5 each). **b** Dermatitis clinical scores and skin biopsy scores for **a**. **c** Immunofluorescence co-localization of MIF and KRT14 in UVB-exposed skin of MRL/lpr mice after injection of sh-CTR AAV or sh-Mif AAV. E, epidermis; D, dermis. **d** Protein levels and quantification of MIF, p-p38, p38, p-C/EBPβ, NLRP3, GSDMD-FL, and GSDMD-NT in epidermal lysates from UVB-exposed skin of MRL/lpr mice after injection of sh-CTR AAV or sh-Mif AAV (*N* = 5 each). **e** Protein levels and quantification of TNFA and MMP9 in epidermal lysates and COL I and MMP2 in dermal lysates from UVB-exposed skin of MRL/lpr mice after injection of sh-CTR AAV or sh-Mif AAV (*N* = 5 each). Data are mean ± SEM. Scale bars = 100 μm. ^*^P < 0.05, ^**^P < 0.01, ^***^P < 0.001.

To evaluate therapeutic potentials of MIF, we generated scramble-shRNA (sh-Ctr) and Mif-shRNA (sh-Mif) AAVs and confirmed the efficacy of sh-Mif AAVs in reducing MIF expression in primary mouse keratinocytes (Fig. S10). Intradermal injection of sh-Mif AAVs prior to UVB irradiation significantly ameliorated skin lesions, reduced dermatitis clinical scores, and improved skin biopsy scores in lupus mice (Fig. 5a, b). The sh-Mif AAVs, but not sh-Ctr AAVs, effectively reduced MIF expression in UVB-exposed keratinocytes (Fig. 5c). Furthermore, analysis of epidermal and dermal lysates from UVB-exposed skin showed that sh-Mif AAVs significantly decreased the levels of p-p38, p-C/EBPβ, NLRP3, and GSDMD-NT in the epidermis, which is predominantly composed of keratinocytes (Fig. 5d). The treatment also reversed the UVB-induced upregulation of TNFA and MMP9 in the epidermis, and COL I and MMP2 in the dermis, which is primarily composed of fibroblasts (Fig. 5e). Collectively, these findings highlight the therapeutic potentials of MIF in UVB-induced skin lesions.

### Transdermal delivery of a MIF inhibitor via microneedle patches attenuated UVB-induced skin lesions in MRL/lpr mice

**Fig. 6:**
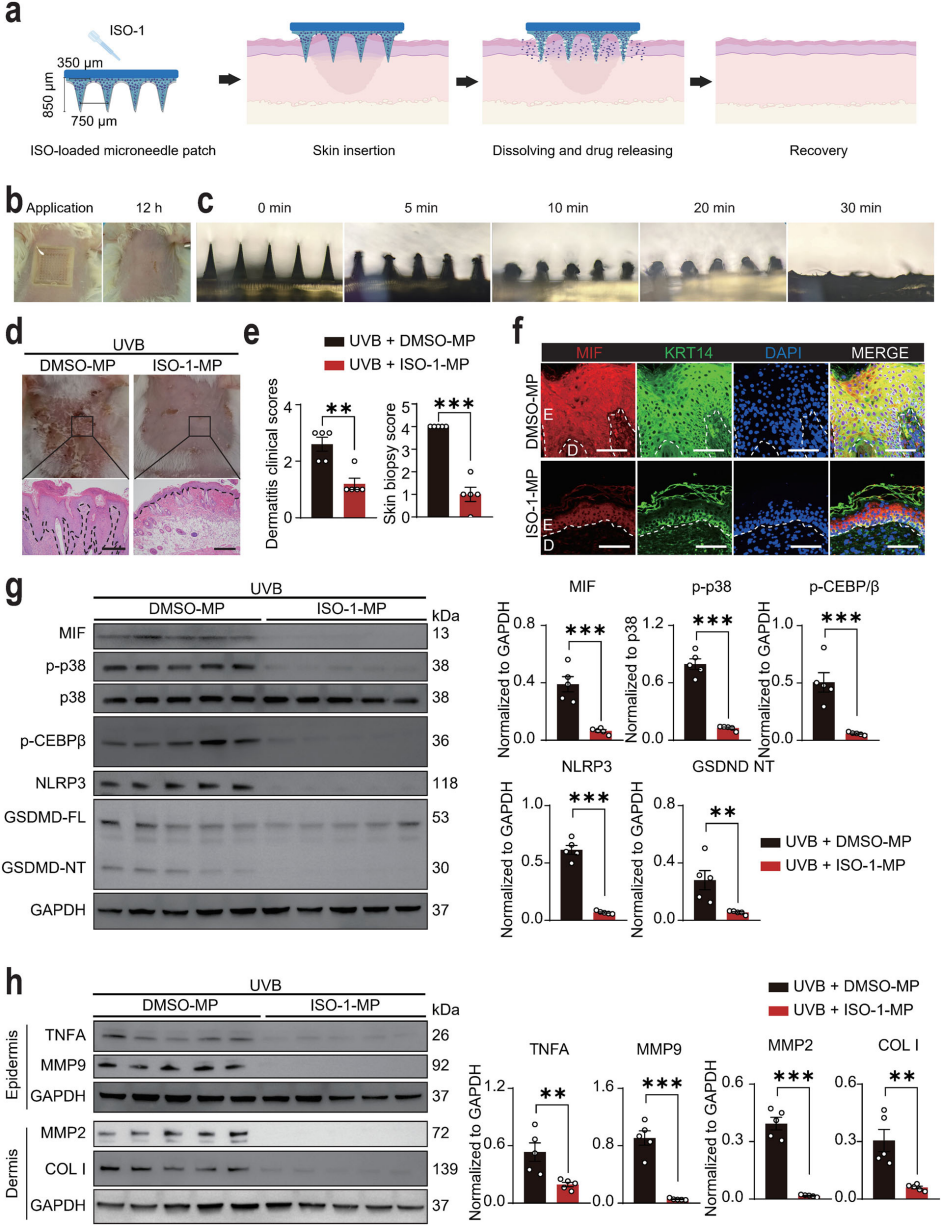
Development of a polymeric microneedle path system for transdermal delivery of MIF inhibitor. **a** Schematic diagram of dissolvable microneedle patch design for MIF inhibitor delivery. **b** Macroscopic appearance of MRL/lpr mouse skin during and 12 h post-microneedle patch application. **c** Degradation kinetics of microneedle patches in rat skin over 5–30 min. **d** Representative macroscopic and H&E-stained images of UVB-exposed skin in MRL/lpr mice treated with DMSO- or ISO-1-loaded microneedle patches (*N* = 5 each). **e** Dermatitis clinical scores and skin biopsy scores for **d**. **f** Immunofluorescence co-localization of MIF and KRT14 in UVB-exposed skin of MRL/lpr mice treated with DMSO- or ISO-1-loaded microneedle patches. E, epidermis; D, dermis. **g** Protein levels and quantification of MIF, p-p38, p38, p-C/EBPβ, NLRP3, GSDMD-FL, and GSDMD-NT in epidermal lysates from UVB-exposed skin of MRL/lpr mice treated with DMSO- or ISO-1-loaded microneedle patches (*N* = 5 each). **h** Protein levels and quantification of TNFA and MMP9 in epidermal lysates and COL I and MMP2 in dermal lysates from UVB-exposed skin of MRL/lpr mice treated with DMSO- or ISO-1-loaded microneedle patches (*N* = 5 each). Data are mean ± SEM. Scale bars = 100 μm. ^**^P < 0.01, ^***^P < 0.001.

To harness the potential of available MIF inhibitors while circumventing their systemic toxicity, we developed a microneedle patch system for the intradermal delivery of the MIF inhibitor ISO-1 (Fig. 6a). The microneedles, arranged in a 10 × 10 array on a 10 × 10 mm patch, exhibited uniform shape and distribution, as confirmed by optical microscopy and scanning electron microscopy (SEM) (Fig. S11a). Mechanical testing demonstrated that the microneedles could withstand a load force of up to 8 N and penetrate to a depth of 450 μm without fracturing, ensuring structural integrity during skin insertion (Fig. S11b). To validate skin penetration, methylene blue-loaded microneedles were applied to porcine skin for 5 min, resulting in a distinct array of blue pinholes at the insertion sites, confirming effective drug delivery (Fig. S11c). Notably, no erythema or edema was observed at the application sites at any time point (Figs. 6b and S11d). Degradation tests revealed that the drug was gradually released within 30 min post insertion (Fig. 6c), underscoring the suitability of the microneedle patch for targeted MIF inhibition.

**Fig. 7:**
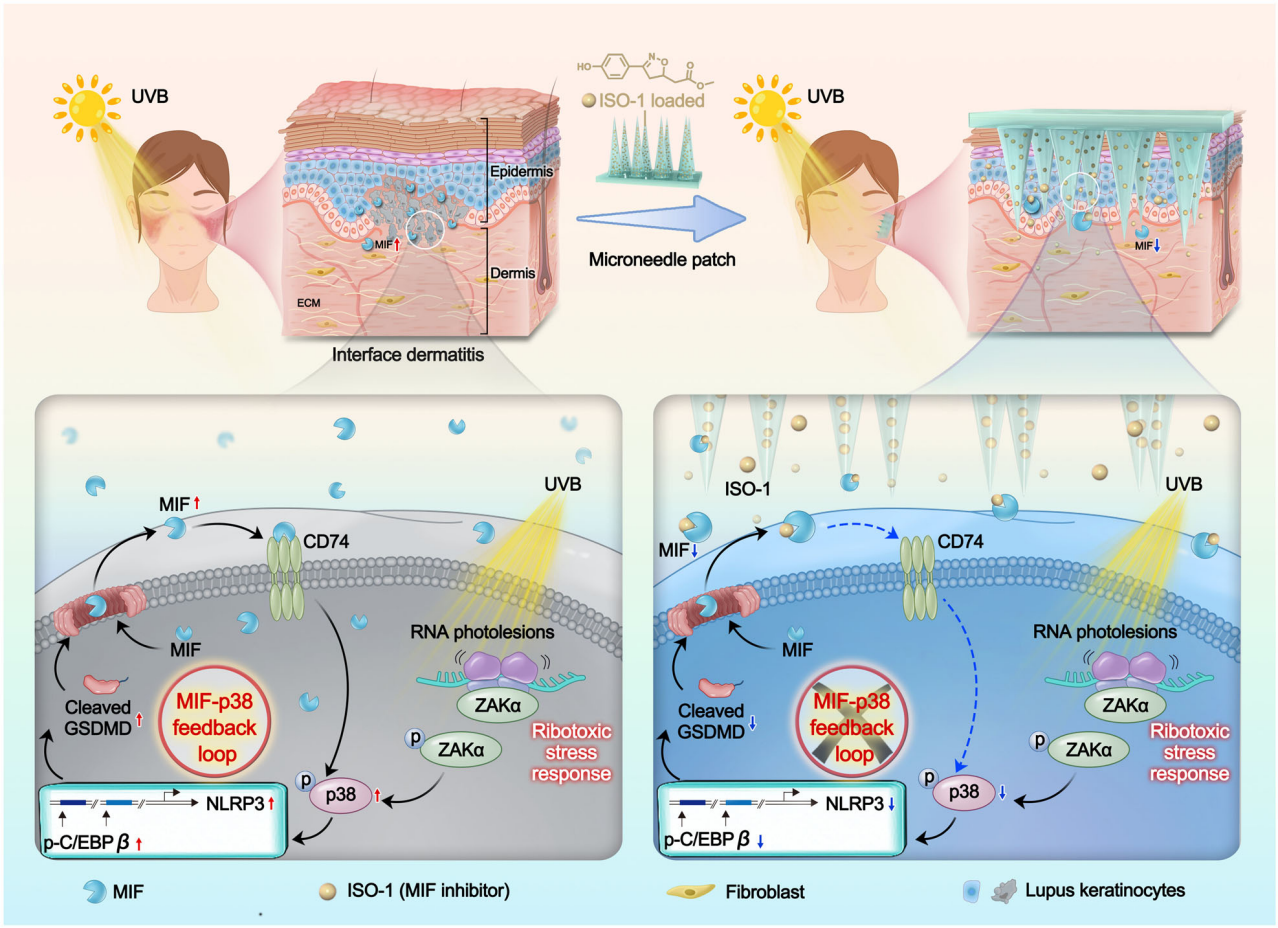
Schematic of UVB-induced CLE pathogenesis and therapeutic intervention. Upon UVB irradiation, keratinocytes initiate a ribotoxic stress response, leading to ZAKα phosphorylation and subsequent p38 activation. p38 upregulates and phosphorylates the transcription factor C/EBPβ, which binds to specific sites on the NLRP3 promoter, driving NLRP3 transcription. Elevated NLRP3 triggers GSDMD cleavage and pyroptotic release of macrophage migration inhibitory factor (MIF). The secreted MIF then binds to its receptor CD74 and reactivates p38 signaling, thereby forming a self-reinforcing feedback loop that sustains NLRP3-GSDMD activation and amplifies inflammation. This positive feedback loop promotes persistent keratinocyte damage and inflammatory pathology in CLE. The therapeutic panel depicts transdermal delivery of the MIF inhibitor ISO-1 via dissolvable microneedle patches, which effectively disrupts the loop and alleviates UVB-induced epidermal hyperplasia, immune infiltration, and cytokine dysregulation in CLE.

Application of the MIF inhibitor-loaded microneedle patch (ISO-1-MP) significantly improved the severity of UVB-induced skin lesions in lupus mice, as evidenced by reduced clinical and biopsy scores compared to the DMSO-MP control group (Fig. 6d, e). While the fluorescence intensity of MIF in individual keratinocytes remained comparable between groups, the overall population of MIF-positive keratinocytes was markedly reduced in the ISO-1-MP group, indicating effective disruption of the MIF-p38-GSDMD feedback loop in lupus keratinocytes (Fig. 6f). Furthermore, protein analysis in separated skin layers demonstrated that ISO-1-MP treatment significantly decreased the levels of MIF, p-p38, p-C/EBPβ, NLRP3, and GSDMD-NT in the epidermis, which is predominantly composed of keratinocytes (Fig. 6g) and reversed the UVB-induced upregulation of TNFA and MMP9 in the epidermis, and COL I and MMP2 in the dermis, which is primarily composed of fibroblasts (Fig. 6h). These findings demonstrate that the microneedle patch system represents a promising, minimally invasive treatment strategy for CLE (Fig. 7). This approach not only addresses the pathological role of keratinocyte-derived MIF but also provides a platform for localized and efficient drug delivery in inflammatory skin diseases.

## Discussion

CLE represents one of the most frequent and earliest clinical manifestations of SLE, highlighting the critical need for early diagnosis and intervention to mitigate systemic progression [2, 4, 5]. UVB is a well-documented environmental trigger for CLE, yet the molecular mechanisms underlying UVB-induced photosensitivity remain incompletely elucidated [4, 13, 14].

In genetically predisposed individuals, even subthreshold UVB exposure can precipitate skin inflammation, suggesting that keratinocytes, as the primary cellular targets of UVB, may harbor intrinsic abnormalities that predispose them to hyperresponsiveness [15, 16, 52]. While prior studies have implicated UVB-induced keratinocyte damage and apoptosis in the accumulation of autoantigens and subsequent immune activation [7], the precise molecular pathways driving photosensitivity in CLE have remained enigmatic. Recent studies have revealed that lupus keratinocytes exhibit aberrant gene expression profiles [10, 11] and release elevated levels of cytokines such as TNF-α and IL-6 following UVB exposure [53–55]. Nevertheless, therapies targeting these cytokines have yielded suboptimal outcomes, suggesting the involvement of other critical mediators [56–58].

Our study provides compelling evidence that keratinocyte-derived macrophage MIF plays a central role in this process by orchestrating a self-amplifying inflammatory loop that drives CLE pathogenesis. MIF, a pleiotropic cytokine with genetic polymorphisms linked to SLE susceptibility [19–21, 23], has been shown to regulate MMPs and cytokines in keratinocytes and fibroblasts [28, 29, 59]. However, its role in CLE had remained unexplored. Through single-cell transcriptomic analysis of human lupus skin, we identified two expanded keratinocyte subpopulations characterized by elevated MIF expression and ISG signatures. These findings were validated in clinical samples across CLE subtypes, where MIF upregulation was consistently observed in the epidermis. Notably, MIF expression was minimally altered in other inflammatory skins, underscoring its specificity to lupus pathogenesis. The identification of these distinct keratinocyte subpopulations provides a cellular basis for understanding the heterogeneity of CLE and suggests that MIF may serve as a key effector molecule linking interferon signaling to tissue inflammation and remodeling. Our study identifies a previously unrecognized inflammatory loop in keratinocytes, thereby elucidating a possible mechanistic link between UVB exposure and the chronic inflammation characteristic of CLE. Our results suggest that keratinocytes are not merely a passive target, but also a central contributor of UVB-mediated lupus skin, a concept that is gaining increasing traction [29, 56].

Our mechanistic investigations revealed that UVB irradiation dose-dependently triggers the release of pre-synthesized MIF from lupus keratinocytes through an RSR-induced p38-GSDMD pyroptosis pathway. This finding is particularly significant as it establishes a direct link between UVB irradiation and MIF release, providing a molecular explanation for photosensitivity in CLE patients. The discovery that MIF release occurs through GSDMD pores during pyroptosis, rather than through conventional secretory pathways, represents a novel mechanism of cytokine release in skin inflammation. This conclusion is strongly supported by our pharmacological evidence demonstrating that MIF release was abolished by the GSDMD pore inhibitor disulfiram but unaffected by inhibitors of vesicular secretion. Furthermore, we demonstrated that released MIF amplifies cutaneous inflammation through autocrine and paracrine signaling, promoting both inflammatory cytokine production and matrix remodeling in keratinocytes and fibroblasts. Our finding that MIF is the dominantly upregulated cytokine in pathogenic keratinocyte subclusters not only aligns with genetic studies linking *MIF* polymorphisms to SLE susceptibility [19–21] but also extends them by pinpointing the specific cellular source and disease context (UVB stress) within the skin.

Previous reports have shown that p38/MAPK enhances the transcriptional activity of C/EBPβ [48, 49], which can regulate NLRP3 expression and its downstream inflammatory responses [50]. Furthermore, MIF plays a critical role in the activation of the NLRP3 inflammasome in both human and mouse models, amplifying inflammatory signaling and pyroptosis [33, 34, 45, 60]. Our data provide direct evidence for the p38-p-C/EBPβ-NLRP3 axis, showing that p38 activation enhances both the expression and phosphorylation of C/EBPβ, which in turn binds to specific sites on the NLRP3 promoter to drive its transcription. The involvement of NLRP3 in this process is particularly noteworthy, as it differs from the established mechanism in normal keratinocytes where UVB primarily activates NLRP1 [31, 37, 38]. This context-dependent switch from NLRP1 to NLRP3 represents a fundamental shift in inflammasome usage in the lupus microenvironment. We propose that in lupus keratinocytes, chronic exposure to eNAs primes the cells, channeling the RSR signal away from canonical NLRP1 proteolytic activation and toward the p38-C/EBPβ-mediated transcriptional upregulation of NLRP3, creating an expanded pool of NLRP3 that becomes the dominant driver of sustained pyroptosis and inflammation. This shift to NLRP3-dependent pyroptosis in lupus keratinocytes may represent an important disease-specific adaptation that contributes to CLE pathogenesis. These findings are further strengthened by recent studies that have established the p38-NLRP3 axis as a critical pathway in cutaneous inflammation [45, 61–63]. For instance, Tang et al. demonstrated in a vitiligo model that stress-related signaling activates p38, which promotes NLRP3 inflammasome activation and pyroptosis, providing a strong parallel to our work in CLE [45]. Chen et al. explicitly identified the p38-NLRP3 pathway in imiquimod-induced skin inflammation, corroborating the broader relevance of this mechanistic axis in chronic inflammatory skin conditions [61].

Our study also demonstrates the therapeutic potential of targeting this inflammatory loop. Both genetic knockdown of MIF using AAV-delivered shRNA and pharmacological inhibition of MIF using microneedle-mediated ISO-1 delivery effectively attenuated UVB-induced skin lesions in lupus-prone mice. The development of a microneedle patch for localized MIF inhibition represents a significant advancement in CLE treatment strategies, offering enhanced skin permeability while minimizing systemic exposure and potential toxicity. This localized approach is supported by the favorable safety profile of ISO-1 observed in previous studies employing systemic administration (40 mg/kg daily) [20, 35], suggesting that our topical delivery strategy should have an excellent tolerability. This approach addresses a critical clinical need for targeted therapies that can effectively manage CLE without the side effects associated with systemic immunosuppression. The limitations of current therapies underscore this need. While conventional systemic agents often lack efficacy in refractory cases, newer biologic therapies have shown variable and often suboptimal outcomes [64]. For instance, B-cell targeting agents like belimumab and rituximab have demonstrated low response rates in CLE [58, 65, 66]. Although litifilimab, which acts on plasmacytoid dendritic cells (pDCs), showed promise, emerging evidence questions the primacy of pDCs as the source of type I interferon in CLE lesions, with keratinocytes being identified as a key producer, which may inherently limit its efficacy [57]. While anifrolumab has shown promising results, targeting the downstream interferon-I receptor may not fully abrogate the inflammation initiated by upstream cellular drivers within the skin, such as keratinocytes [32, 67]. Collectively, the limitations of these therapies highlight the need for strategies that directly target the core pathogenic circuits within the cutaneous microenvironment, particularly those driven by keratinocytes. Our approach, focusing on the keratinocyte-centric MIF-p38-GSDMD loop, is distinct from and complementary to existing immune-centric models.

In conclusion, our findings not only advance our understanding of CLE pathogenesis but also identify MIF as a promising therapeutic target. By situating our findings within the context of existing literature on UVB responses, inflammasome biology, p38 signaling, and the current therapeutic landscape, we highlight the significance and novelty of the MIF-p38-GSDMD axis. The development of microneedle-based delivery systems for MIF inhibition offers a clinically viable strategy for managing this challenging condition. Future studies exploring the efficacy of MIF-targeted therapies in human CLE patients are warranted to translate these findings into clinical practice.

## Materials and methods

### Ethics and study design

All animal experiments were approved by the Institutional Animal Care and Use Committee (IACUC) of Sun Yat-Sen University (Approval No. SYSU-IACUC-2024-000943). All human studies were approved by the Institutional Review Board of Sun Yat-Sen Memorial Hospital, Sun Yat-sen University (Approval No. SYSKY-2023-745-01), and written informed consent was obtained from all participants. All methods were performed in accordance with the relevant guidelines and regulations. For in vivo studies, sample sizes were chosen based on established practices in the field and our prior experience. No statistical method was used to predetermine sample size, but our sample sizes are similar to those reported in previous publications. Mice were randomly assigned to experimental groups. Investigators were blinded to group allocation during outcome assessment. No data were excluded from the analysis. No specific inclusion or exclusion criteria were pre-established beyond the diagnosis of CLE or normal control status for human samples, and healthy status for animal studies.

### Animal experiments

Female MRL/lpr mice (12 weeks old) were procured from Jiangsu Huachuang Xinnuo Medical Technology Co., Ltd. Mice were housed in a specific pathogen-free facility under controlled conditions (12:12 light-dark cycle, 60–80% humidity, 22 ± 1 °C) with no more than five animals per cage.

### UVB irradiation

UVB irradiation was performed using an SS-01 instrument (SIGMA, Shanghai) with a wavelength range of 290–320 nm (peak at 311 nm) and an irradiance of 11.00 mW/cm². For animal experiments, a 1.5 cm diameter area on the shaved dorsal skin was exposed to UVB in four cycles (5 days irradiation followed by 2 days’ rest). The irradiation energy was 330 mJ/cm² for the first two cycles and 110 mJ/cm² for the latter two cycles (Table S1). Skin lesion severity was assessed using established clinical and histopathological scoring systems [68, 69].

### AAVs production and injection

Recombinant AAVs were produced by packaging lentiviruses in HEK293T cells using a three-plasmid system, which included a packaging plasmid, a helper plasmid, and a transfer plasmid. The double-stranded AAV serotype 2/9 (dsAAV2/9) was utilized for this study. The cytomegalovirus (CMV) promoter was employed to drive gene expression, and the enhanced green fluorescent protein (EGFP) was incorporated as a reporter gene. The targeted sequence for Mif was CCGCAACTACAGTAAGCTG, while the control sequence was CCTAAGGTTAAGTCGCCCTCG.

For in vivo experiments, MRL/lpr mice were anesthetized, and a subcutaneous injection was administered in the shaved dorsal skin area. Each mouse received a viral load of 1.0 × 10^11^ genomic copies of pscAAV-U6-shRNA (Mif)-CMV-EGFP-tWPA in a 100 µL volume. Control mice were injected with an equal volume of pscAAV-U6-shRNA (NC2)-CMV-EGFP-tWPA. One-week post-injection, the mice were used for subsequent experiments to evaluate the therapeutic efficacy of the MIF-targeted intervention.

### Clinical samples

CLE diagnosis was confirmed through clinical and histopathological evaluation [70], while SLE classification adhered to the 2019 EULAR/ACR criteria [12]. Skin tissues from patients with ACLE, SCLE, and CCLE were included for immunohistochemical analysis of MIF expression. Patients with concurrent malignancy or active infection were excluded. Normal skin tissues were obtained from UVB-unexposed sites adjacent to excised pigmented nevi. Lesional and normal tissues were age- and sex-matched. Detailed donor information and tissue origins are provided in Table S2.

### Cells culture and treatment

Human immortalized epidermal keratinocytes (HaCaT cells) were obtained from iCell Bioscience Inc. (Shanghai, China). The cell line was authenticated by short tandem repeat (STR) profiling and tested negative for mycoplasma contamination. Primary fibroblasts were isolated from skin tissues as previously described [71]. The cells were cultured in Dulbecco’s Modified Eagle Medium (DMEM; GNM12800, Genom, Hangzhou, China) supplemented with 10% fetal bovine serum (FSP500, ExCell Bio, Shanghai, China), 100 μ/mL penicillin, and 100 μg/mL streptomycin (SV30010, HyClone, Logan, UT) at 37 °C in a humidified 5% CO_2_ incubator. Cells were harvested using 0.25% trypsin/EDTA solution (25200072, Gibco, Grand Island, NY) for subculturing or downstream experiments.

For stimulation or inhibition assays, cells were treated under specific conditions. To study the effects of MIF, cells were cultured in medium containing 100 ng/mL recombinant MIF (BK0133, bioworld, Nanjing, China) for 4 h [47]. To inhibit MIF activity, cells were treated with 25 μM ISO-1 (I303791, Aladdin, Shanghai, China) for 24 or 48 h [33, 35]. To activate the ZAKα pathway, cells were exposed to 1 μM anisomycin (ANS, HY-18982, MCE) for 3 h [37]. For pathway inhibition studies, cells were pretreated with 10 μM SB203580 (S1863, Beyotime, Shanghai, China) or 5 μM MCC950 (CP-456773, Selleck) for 2 h prior to UVB exposure [72, 73]. To investigate MIF release mechanisms, cells were exposed to UVB in the presence of 40 μM disulfiram (DSF, a GSDMD pore inhibitor, MCE), 10 μM GW4869 (an exosome inhibitor, APExBIO), or 5 μg/mL brefeldin A (a Golgi-mediated secretion inhibitor, APExBIO) [74–76].

### Cell transfection

HaCaT cells or primary mouse keratinocytes (iCell, Shanghai, China) were seeded in 6-well plates and cultured until they reached 60–80% confluence. Transfection was performed using Lipofectamine 2000 (11668-019, ThermoFisher) according to the manufacturer’s instructions. Briefly, the transfection mixture was prepared by combining siRNA with Lipofectamine 2000 in Opti-MEM medium and incubated at room temperature for 20 min. The mixture was then added to the cells, which were incubated at 37 °C for 8 h. After incubation, the transfection medium was replaced with complete culture medium, and the cells were further cultured for 24 h for quantitative real-time PCR (qPCR) analysis or 48 h for Western blot (WB) analysis. The siRNA sequences used for transfection were synthesized by GenePharma (Suzhou, China) are provided in Table S3.

### Endogenous nucleic acids extraction and transfection

Cytosolic DNA and RNA were extracted from HaCaT cells using the DNA Extractor Kit (BioTeke Corporation, Beijing, China) and the EZ-press RNA Purification Kit (B0004DP, EZBioscience, Roseville, MN), respectively, following the manufacturers’ instructions. The extracted eNAs were subsequently transfected into recipient HaCaT cells using Lipofectamine 2000, as previously described [40, 41]. To validate the lupus-like phenotype, transcript levels of interferon-stimulated genes (*ISG15*, *IFI44*, *RIGI*, *MX1*, *OAS1*, *OASL*, *IFI44L*, *IFI27*) and keratinocyte subcluster markers (*CXCL10*, *IFITM1*, *S100A8*, *S100A9*) were quantified by qPCR in eNAs-transfected cells.

### Chromatin immunoprecipitation (ChIP) assay

ChIP assay was performed using the Beyotime ChIP Kit (P2083S). HaCaT cells in 10-cm dishes were crosslinked with 1% formaldehyde (37 °C, 10 min), quenched with glycine solution (5 min), and harvested in PBS containing protease inhibitors. Nuclei from ~4 × 10⁶ cells were isolated in 1X Buffer A, digested with MNase (1 µl, 2000 gel units, 37 °C, 20 min) in 1X Buffer B, and sonicated to disrupt membranes. Chromatin was diluted in ChIP Buffer and immunoprecipitated overnight (4 °C) with 1 µg anti-C/EBPβ (Proteintech) or control IgG. Complexes were captured with Protein A/G Magnetic Beads, washed sequentially with kit-specific buffers (Low/High Salt, LiCl, TE), and eluted. DNA was reverse-crosslinked (65 °C, 4 h; 0.3 M NaCl), protease K-treated, and purified. NLRP3 promoter enrichment at three sites was quantified by qPCR using the following primers:

Fragment 1-F: 5′-GTGCCAAGTTTTATTCTTTTGCGTG-3′

Fragment 1-R: 5′-CAAGGTCACCAAGAAGACACAATG-3′

Fragment 2-F: 5′-GGAGTCTTGCTCTTGTCACC-3′

Fragment 2-R: 5′-GAGAATCACTTGAACCTGGGAG-3′

Fragment 3-F: 5′-GTCATCGGAACCAACTGCTTATTCT-3′

Fragment 3-R: 5′-GACCAAGCACTACATCAACCCAAA-3′

### Dual Luciferase Reporter Assay

To investigate C/EBPβ-mediated transcriptional activation of the NLRP3 promoter, HaCaT cells were seeded in 24-well plates (70% confluency) 24 h prior to transfection. Different fragments of the human NLRP3 promoter sequence were cloned into the pGL3-Basic firefly luciferase reporter vector. Cells were co-transfected with 400 ng of the NLRP3-promoter construct and 40 ng of the Renilla luciferase control plasmid using Lipofectamine 3000 (Invitrogen).

After 48 h, cells were lysed with 150 μl of ice-cold lysis buffer (Dual Luciferase Reporter Gene Assay Kit, Yeasen Biotechnology, Cat# 11402E560) per well. Lysates were centrifuged (12,000 × *g*, 1 min, 4 °C), and 20 μL of supernatant was transferred to opaque 96-well plates. Firefly luciferase activity was measured immediately after injecting 100 μl of Firefly Luciferase Working Solution. Renilla luciferase activity was then quantified following addition of 100 μL Renilla Luciferase Working Solution using a microplate reader. Relative promoter activity was calculated as the Firefly/Renilla luminescence ratio.

### Quantitative real-time PCR (qRT-PCR)

Total RNA was extracted from HaCaT cells, fibroblasts, and skin tissues using the EZ-press RNA Purification Kit (B0004DP, EZBioscience, Roseville, MN) following the manufacturer’s instructions. cDNA synthesis was performed using the Color Reverse Transcript Kit (A0010CGQ, EZBioscience, Roseville, MN). qRT-PCR was carried out on a Roche LightCycler® 480 instrument using SYBR Green Color qPCR Mix (A0012-R2, EZBioscience, Roseville, MN).

The comparative threshold cycle (Ct) method was used to quantify gene expression levels. The relative mRNA expression of target genes was normalized to the expression of housekeeping genes, either *GAPDH* or *Actb*. Gene-specific primer sequences used in the qRT-PCR assays are listed in Table S4.

### Western blot analysis

Whole-cell lysates were prepared using radioimmunoprecipitation assay lysis buffer (CW2333S, CWBIO, Jiangsu, China) supplemented with protease and phosphatase inhibitors (P1045, Beyotime, Shanghai, China) to ensure complete protein extraction. Protein concentrations were quantified using a bicinchoninic acid assay (23228, ThermoFisher). For the analysis of epidermal and dermal protein expression, mouse skin tissues were separated into their constituent layers. Briefly, full-thickness skin biopsies were dissected into 3–5 mm pieces and incubated in Dispase II solution (10 mg/mL, Roche) prepared in Hanks’ Balanced Salt Solution with Ca²⁺/Mg²⁺ (Sigma-Aldrich) at 37 °C for 1 h. Following enzymatic treatment, the epidermis was carefully peeled from the dermis using fine forceps. Both the epidermal and dermal tissues were collected separately for protein extraction and immunoblotting. For immunoblot analysis, primary antibodies targeting specific proteins, including Anti-MIF, Anti-Collagen I, Anti-MMP2, Anti-MMP9, Anti-TNF alpha, Anti-IL-1 beta, Anti-MX1, Anti-p38, Anti-p-p38, Anti-ZAKα, Anti-GSDMD, Anti-GSDME, Anti-NLRP1, Anti-NLRP3, Anti-C/EBPβ, Anti-p-C/EBPβ, and Anti-GAPDH, were used according to the manufacturers’ protocols.

Phosphorylated ZAKα was detected using a Phos-tag 7.5% SDS-PAGE gel (198-17981, Wako, Tokyo, Japan). Protein bands were visualized using a chemiluminescence ECL substrate (WBKLSO100, Merck Millipore, Billerica, MA), and the signal intensities were quantified using ImageJ software. Detailed information on the antibodies is provided in Table S5.

### Enzyme-linked immunosorbent assay (ELISA)

HaCaT cells were cultured in 12-well plates until they reached 80% confluence. The culture medium was collected and centrifuged to remove cellular debris. The levels of macrophage MIF in the supernatant were quantified using a Human MIF ELISA Kit (70-EK1158, MultiSciences, China) according to the manufacturer’s instructions. Briefly, standards and samples were added to the pre-coated wells and incubated with detection reagents. After washing, the optical density (O.D.) was measured at 450 nm using an ELISA analytical instrument (Molecular Devices, SpectraMax® Plus 384). The concentration of MIF in the samples was determined by comparing the O.D. values to a standard curve generated from known concentrations of recombinant MIF.

### Immunohistochemistry

Paraffin-embedded skin tissue sections were deparaffinized using xylene and rehydrated through a graded series of ethanol solutions. Heat-induced epitope retrieval was performed to unmask antigenic sites. The sections were then blocked with goat serum to reduce nonspecific binding and incubated overnight at 4 °C with primary antibodies, including rabbit anti-human MIF and rabbit anti-human CD74. Following primary antibody incubation, the sections were stained using a horseradish peroxidase (HRP)-conjugated secondary antibody, and the staining was visualized using a 3,3′-diaminobenzidine (DAB) kit (ZLI-9017, ZS, Beijing, China).

Quantitative analysis of the staining was performed using Image-Pro Plus 6.0 software (Media Cybernetics) [77]. The integrated optical density (IOD), defined as the total count of positive staining across all locations within the region of interest, was measured. The mean density was calculated as the ratio of IOD to the selected area (IOD/Area). For CLE subtype analysis, MIF expression was quantified in the epidermis of normal skin and ACLE, SCLE, and CCLE lesions. Detailed information on the antibodies is provided in Table S5.

### Immunofluorescence

Tissue sections were incubated overnight at 4 °C with a mixture of two primary antibodies, including rabbit anti-human/mouse MIF paired with mouse anti-human/mouse KRT14, or rabbit anti-human CD74 paired with mouse anti-human KRT14 or mouse anti-human vimentin. Following primary antibody incubation, the sections were washed and incubated for 1 h at room temperature with a mixture of secondary antibodies: goat anti-mouse IgG H&L conjugated to Alexa Fluor® 488 and goat anti-rabbit IgG H&L conjugated to Alexa Fluor® 555, diluted in blocking buffer.

After secondary antibody incubation, the sections were washed and mounted using glycerol mounting media supplemented with anti-fade reagent and DAPI (ab188804, Abcam, Cambridge, UK) to counterstain nuclei. The slides were visualized using a fluorescence microscope (Olympus IX73, Japan). Detailed information on the antibodies is provided in Table S5.

### Single-cell RNA sequencing (scRNA-seq) analysis

Single-cell RNA sequencing (scRNA-seq) data were obtained from the Gene Expression Omnibus (GEO) under accession number GSE186476. The dataset included 14 normal skin samples and 7 paired samples of non-lesional (from sun-protected buttock skin) and lesional skin from lupus patients. Data analysis was performed using the Seurat V3.0 R package. The raw datasets were integrated, normalized, and subjected to dimensionality reduction using principal component analysis. Cell clustering was conducted based on highly variable genes, and the resulting clusters were visualized using UMAP or t-distributed stochastic neighbor embedding (t-SNE) at a clustering resolution of 0.5.

### Fabrication and characterization of microneedle patches

Microneedle patches were fabricated using a polydimethylsiloxane (PDMS) micromold, with each microneedle measuring 350 μm in base length and 850 μm in height. The needles were arranged in a 10 × 10 array with a needle pitch of 750 μm. A polyvinylpyrrolidone (PVP) solution was prepared and mixed with either ISO-1 (100 mM) or a DMSO control solution. The mixture was centrifuged for 20 min to ensure homogeneity. The resulting solution was then injected into the microneedle mold and subjected to vacuum treatment to remove air bubbles, followed by overnight drying. After drying, the PVP solution was reintroduced into the mold to form the patch base and allowed to dry overnight. The final concentration of ISO-1 in each patch was ~235.4 μg.

Following fabrication, the microneedle patches were carefully removed from the mold, and their morphology was characterized using an optical microscope (Olympus CKX-41-32, Japan) and a scanning electron microscope (PHENOMPURE, Thermo Fisher, USA).

### Microneedle compression test and skin insertion test

The mechanical strength of the microneedles was evaluated using a universal testing machine (PT-1699V, Baoda, China). For compression testing, the microneedle patch was positioned at the center of the testing base with the needle tips facing upward. A vertical force was applied at a constant rate of 0.2 mm/min until a displacement of 450 μm was achieved. The trigger force was set at 0.05 N, and the force-displacement curve was recorded to assess the mechanical properties of the microneedles.

To evaluate skin insertion capability, the microneedle patches were soaked in a methylene blue solution and pressed onto porcine skin with a controlled pressure of 5–10 N for 5 min. After removal, the insertion sites were examined using an optical microscope (Olympus CKX-41-32, Japan) to visualize and document the penetration patterns.

### Microneedle degradation test and skin irritation test

To assess the degradation kinetics of the microneedles, the abdominal skin of anesthetized rats was shaved, and the microneedle patch was firmly pressed onto the skin and secured with medical tape. The patches were removed at intervals of 5-, 10-, 20-, and 30-min post application. The insertion sites were then photographed using an optical microscope (Olympus CKX-41-32, Japan) to evaluate the extent of microneedle dissolution and skin penetration over time.

For the skin irritation test, the microneedle patch was applied vertically to the shaved abdominal skin of rats and maintained in place for 30 min. After removal, the skin was monitored for signs of irritation, including erythema (redness) and edema (swelling), at 0, 1, 6, 12, and 24 h. The severity of these reactions was documented to evaluate the biocompatibility and safety of the microneedle patches.

### Statistical analysis

All data are presented as mean ± standard error of the mean (SEM) and were analyzed using GraphPad Prism version 8.0 (GraphPad Software, San Diego, CA, USA). The normality of data distribution was assumed based on the extensive use of these parametric tests in analogous experimental settings within the field. For parametric comparisons between two groups, an unpaired *t*-test was applied, while the Mann–Whitney U test was used for non-parametric comparisons. For analyzes involving multiple groups, one-way analysis of variance (ANOVA) followed by Dunnett’s multiple comparisons test was performed. For comparisons involving multiple factors, two-way ANOVA followed by Tukey’s multiple comparisons test was performed. All statistical tests were two-sided, and adjustments for multiple comparisons were applied where appropriate. Error bars in figures represent SEM unless otherwise specified. A *P*-value of less than 0.05 was considered statistically significant.

## Supplementary information

Supplementary Materials

Fig. S1

Fig. S2

Fig. S3

Fig. S4

Fig. S5

Fig. S6

Fig. S7

Fig. S8

Fig. S9

Fig. S10

Fig. S11

Fig. S12

Full scans of Western blots

## Data Availability

All data generated or analyzed during this study are included in this published article and its supplementary information files. This study involved the re-analysis of publicly available single-cell RNA sequencing data from GEO under accession number GSE186476. All of the data can be found in either the main text or the supplementary materials.
