# Supplementary material for: A MIF-p38-GSDMD inflammatory loop in keratinocytes underlies UVB-induced cutaneous lupus
Source: Cell Death Dis. 2026 Feb 2;17(1):198. doi: 10.1038/s41419-026-08443-4 (PMC12876878; doi:10.1038/s41419-026-08443-4)
Supplement: Supplementary file 1 — Supplementary Materials [file 41419_2026_8443_MOESM1_ESM.docx]

# Supplementary Materials

| Intensity (mJ/cm^2^) | Day 1 | Day 2 | Day 3 | Day 4 | Day 5 | Day 6 | Day 7 |
| --- | --- | --- | --- | --- | --- | --- | --- |
| Week 1 | 330 | 330 | 330 | 330 | 330 | / | / |
| Week 2 | 330 | 330 | 330 | 330 | 330 | / | / |
| Week 3 | 110 | 110 | 110 | 110 | 110 | / | / |
| Week 4 | 110 | 110 | 110 | 110 | 110 | / | / |

**Table S1.** The UVB exposure protocols for MRL/lpr mice.

**Table S2.** The demographics and characteristics of healthy controls, DM, LSA and CLE patients.

| **Tissues** | **Sex** | **Age, y** | **Site** |
| --- | --- | --- | --- |
| Normal skin | M | 30 | Limb |
|  | F | 26 | Limb |
|  | F | 24 | Trunk |
|  | M | 59 | Limb |
|  | F | 34 | Trunk |
|  | M | 35 | Trunk |
|  | F | 62 | Limb |
| DM | F | 84 | Neck |
|  | M | 41 | Face |
|  | M | 77 | Back |
|  | F | 42 | Face |
|  | F | 51 | Trunk |
| LSA | F | 9 | Vulva |
|  | F | 34 | Vulva |
|  | F | 22 | Trunk |
|  | F | 32 | Vulva |
|  | M | 34 | Back |
| CLE | M | 62 | Limb |
|  | M | 55 | Limb |
|  | M | 48 | Face |
|  | M | 66 | Limb |
|  | F | 36 | Neck |
|  | F | 69 | Scalp |
|  | F | 24 | Scalp |
|  | F | 30 | Limb |
|  | F | 57 | Face |
|  | M | 56 | Chest |
|  | M | 58 | Trunk |
|  | F | 27 | Face |

Abbreviations: M, male; F, female.

**Table S3**. List of siRNA and shRNA sequences.

| **Symbol** | **sense** | **anti-sense** |
| --- | --- | --- |
| MIF-siRNA #1 | UGGUGUUUACGAUGAACAUTT | AUGUUCAUCGUAAACACCATT |
| MIF-siRNA #2 | UUGGUGUUUACGAUGAACAUCGGCA | UGCCGAUGUUCAUCGUAAACACCAA |
| CD74-siRNA #1 | AUCCAUGACUGGCUUCUGAUCUUCC | GGAAGAUCAGAAGCCAGUCAUGGAU |
| CD74-siRNA #2 | GGCUGGACAAACUGACAGUTT | ACUGUCAGUUUGUCCAGCCTT |
| Mif-shRNA | CCGCAACTACAGTAAGCTG | CAGCTTACTGTAGTTGCGG |

**Table S4.** List of primers for qRT-PCR.

|  | | **Symbol** | **Forward** | **Reverse** |
| --- | --- | --- | --- | --- |
| mouse | |  |  |  |
|  | | Mif | GAGGGGTTTCTGTCGGAGC | GTTCGTGCCGCTAAAAGTCA |
|  | Actb | | GTGACGTTGACATCCGTAAAGA | GCCGGACTCATCGTACTCC |
| human |  | |  |  |
|  | MIF | | ACCAGCTCATGGCCTTCG | CTTGCTGTAGGAGCGGTT |
|  | CD74 | | AGGGCCTGGACATTGACCA | CACCTACAGACCACTTCACTTC |
|  | CD44 | | CTGCCGCTTTGCAGGTGTA | CATTGTGGGCAAGGTGCTATT |
|  | TNFA | | CCTCTCTCTAATCAGCCCTCTG | GAGGACCTGGGAGTAGATGAG |
|  | IL1B | | ATGATGGCTTATTACAGTGGCAA | GTCGGAGATTCGTAGCTGGA |
|  | IFNG | | TCGGTAACTGACTTGAATGTCCA | TCGCTTCCCTGTTTTAGCTGC |
|  | MX1 | | GTTTCCGAAGTGGACATCGCA | CTGCACAGGTTGTTCTCAGC |
|  | COL1A1 | | GAGGGCCAAGACGAAGACATC | CAGATCACGTCATCGCACAAC |
|  | MMP2 | | TACAGGATCATTGGCTACACACC | GGTCACATCGCTCCAGACT |
|  | MMP9 | | TGTACCGCTATGGTTACACTCG | GGCAGGGACAGTTGCTTCT |
|  | GAPDH | | GGAGCGAGATCCCTCCAAAAT | GGCTGTTGTCATACTTCTCATGG |
|  | ISG15 | | CGCAGATCACCCAGAAGATCG | TTCGTCGCATTTGTCCACCA |
|  | IFI44 | | ATGGCAGTGACAACTCGTTTG | TCCTGGTAACTCTCTTCTGCATA |

**Table S5.** List of antibodies.

| **Antibody** | **Company** | **Clone** | **Assay** |
| --- | --- | --- | --- |
| anti-MIF | Abcam | ab187064 | IHC; IF; WB |
| anti-CD74 | Abcam | ab108393 | IHC; IF; WB |
| anti- KRT14 | Proteintech Group | 60320-1-Ig | IF |
| anti- vimentin | Abcam | ab8978 | IF |
| anti-mouse IgG H&L (Alexa Fluor ®488) | Abcam | ab150113 | IF |
| anti-Rabbit IgG H&L (Alexa Fluor® 555) | Abcam | ab150078 | IF |
| anti-Collagen I | Abcam | ab260043 | WB |
| anti-MMP2 | CST | 87809S | WB |
| anti-MMP9 | Abcam | ab283575 | WB |
| anti-TNF alpha | Abcam | ab1793 | WB |
| anti-IL-1 beta | Abcam | ab283818 | WB |
| anti-MX1 | Abcam | ab207414 | WB |
| anti-p38 | CST | 8689 | WB |
| anti-p-p38 | CST | 4511T | WB |
| anti-ZAK | Proteintech Group | 28761-1-AP | WB |
| anti-GSDMD | CST | 39754S | WB |
| anti-NLRP3 | Invitrogen | MA5-23919 | WB |
| anti-GAPDH | Abcam | ab8245 | WB |
| anti-C/EBPβ | ABclonal | A0711 | WB |
| anti-p-C/EBPβ | ABclonal | AP1055 | WB |

**Figure S1. MIF expression is elevated across CLE subtypes.**

Quantification of MIF expression in normal control (NC, N = 4) and different subtypes of cutaneous lupus erythematosus (CLE) skin tissues, including acute cutaneous lupus erythematosus (ACLE, N = 4), subacute cutaneous lupus erythematosus (SCLE, N = 4), and chronic cutaneous lupus erythematosus (CCLE, N = 4).

Data are mean ± SEM. **P* < 0.05.

**Fig. S2.** **Expression of MIF signaling, inflammatory cytokines, and tissue remodeling markers in skin tissues.**

**(a)** mRNA expression levels of *MIF*, *CD74*, *CD44*, *COL1A1*, *MMP2*, *MMP9*, *IL1B*, *TNFA*, *IFNG*, and *MX1* in skin tissues from normal controls (NC, N = 3), dermatomyositis (DM, N = 5), lichen sclerosus et atrophicus (LSA, N = 5), and cutaneous lupus erythematosus (CLE, N = 6).

Data are mean ± SEM. **P* < 0.05, ***P* < 0.01, ****P* < 0.001.

**(b)** Correlation analysis between *MIF* mRNA expression and the expression of *TNFA*, *IL1B*, *IL6*, *MMP2*, *MMP9*, *COL1A1*, *MX1*, and *IFNK* in human skin tissues from normal and lupus lesional skin samples. Each scatter plot represents a separate correlation analysis performed using simple linear regression; *P*-values and coefficients of determination (R²) are indicated.

**Fig. S3. UVB exposure does not alter *MIF* mRNA levels in keratinocytes.**

**(a)** mRNA levels of *MIF* in HaCaT cells 24 hours post-UVB exposure at varying intensities (11–165 mJ/cm²) (N = 4).
**(b)** mRNA levels of *MIF* in HaCaT cells exposed to UVB (55 mJ/cm²) for 6, 12, and 24 hours (N = 4).

Data are mean ± SEM.

**Figure S4. Pharmacological inhibition of MIF attenuates UVB-induced inflammatory responses.**

**(a-b)** Protein levels of MMP9 and TNFA in keratinocytes **(a)** and COL I and MMP2 in fibroblasts **(b)** treated with conditioned media from UVB-irradiated keratinocytes in the presence or absence of MIF inhibitor ISO-1 (50 μM).

Data are mean ± SEM. **P* < 0.05, ****P* < 0.001.

**Figure S5. eNAs-transfected keratinocytes recapitulate key features of lupus keratinocytes.**

mRNA expression of interferon-stimulated genes (*ISG15*, *IFI44*, *RIGI*, *MX1*, *OAS1*, *OASL*, *IFI44L*, *IFI27*) and markers of pathogenic keratinocyte subpopulations (*CXCL10*, *IFITM1*, *S100A8*, *S100A9*) in HaCaT cells transfected with eNAs or control.

Data are mean ± SEM. **P* < 0.05, ***P* < 0.01, ****P* < 0.001.

**Figure S6. RSR specifically activates GSDMD but not GSDME in lupus keratinocytes.**

Western blot analysis of GSDME-FL and GSDME-NT in eNAs-transfected HaCaT cells treated with UVB or anisomycin (ANS).

Data are mean ± SEM. ****P* < 0.001.

**Figure S7. UVB irradiation does not alter NLRP1 cleavage in lupus keratinocytes.**

Western blot analysis of NLRP1-FL and NLRP1-NT in eNAs-transfected HaCaT cells following UVB irradiation.

Data are mean ± SEM.

**Figure S8. Genetic knockdown of NLRP3 attenuates UVB-induced GSDMD cleavage.**

Western blot analysis of NLRP3, GSDMD-FL, and GSDMD-NT in eNAs-transfected HaCaT cells transfected with si-CTR or si-NLRP3 followed by UVB irradiation.

Data are mean ± SEM. ***P* < 0.01, ****P* < 0.001.

**Fig. S9. UVB-induced skin lesions in lupus-prone MRL/lpr mice highlight MIF signaling activation and associated pathological remodeling.**

**(a)** Schematic diagram of the experimental design for establishing a UVB-induced skin lesion model in lupus-prone MRL/lpr mice.

**(b)** Representative macroscopic and H&E-stained images of untreated and UVB-exposed skin in MRL/lpr mice (N = 4 each).

**(c)** Dermatitis clinical scores and skin biopsy scores for **(b)**.

**(d)** Immunofluorescence co-localization of MIF and KRT14 in untreated and UVB-exposed skin of MRL/lpr mice.

**(e)** Protein levels and quantification of COL I, MMP9, MMP2, and TNFA in untreated and UVB-exposed skin of MRL/lpr mice (N = 4 each).

**(f)** Protein levels and quantification of MIF, p-p38, p38, NLRP3, GSDMD-FL, and GSDMD-NT in untreated and UVB-exposed skin of MRL/lpr mice (N = 4 each).

Data are mean ± SEM. **P* < 0.05, ****P* < 0.001.

**Fig. S10. Efficient knockdown of *Mif* in primary mouse keratinocytes.**

mRNA levels of *Mif* in primary mouse keratinocytes transfected with control-shRNA or *Mif*-shRNA (N = 4).

Data are mean ± SEM. ****P* < 0.001.

**Fig. S11.** **Development and functional evaluation of MIF inhibitor ISO-1-loaded microneedle patches for cutaneous delivery.**

**(a)** Representative stereo-microscopic and SEM images of microneedle patches.

**(b)** Mechanical strength characterization of DMSO- and ISO-1-loaded microneedle patches (MP).

**(c)** Skin insertion tests of DMSO- and ISO-1-loaded microneedle patches stained with methylene blue.

**(d)** Representative images of rat skin after microneedle patch insertion for 30 minutes, photographed at 0, 1-, 6-, 12-, and 24-hours post-removal.

**Fig. S12. Full scans of Western blots.**

Uncropped western blot images corresponding to the figures in the text.
