# Supplementary figures and images for: A MIF-p38-GSDMD inflammatory loop in keratinocytes underlies UVB-induced cutaneous lupus
Source: Cell Death Dis. 2026 Feb 2;17(1):198. doi: 10.1038/s41419-026-08443-4 (PMC12876878; doi:10.1038/s41419-026-08443-4)

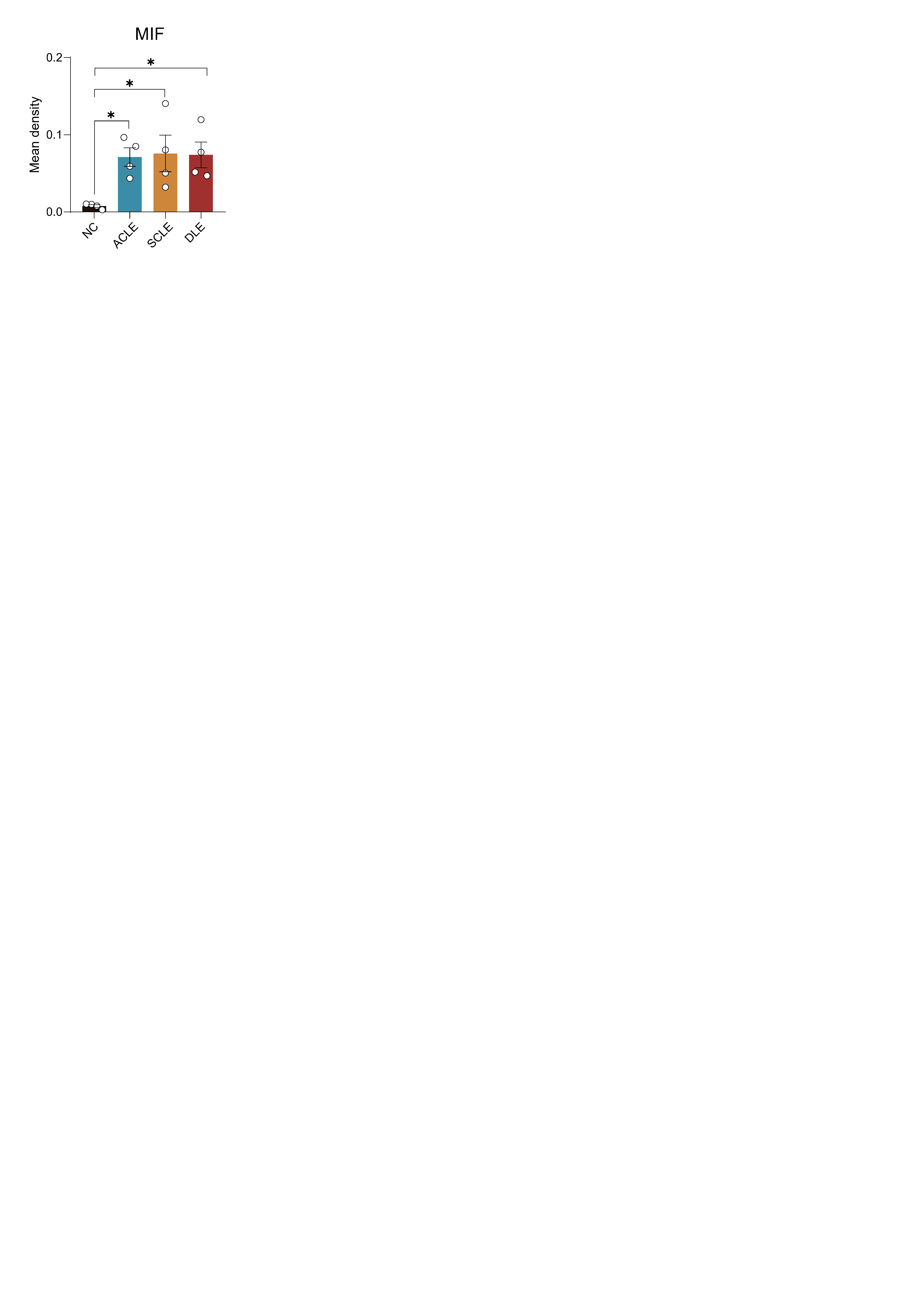

Supplement: Supplementary file 2 — Fig. S1 [file 41419_2026_8443_MOESM2_ESM.jpg]

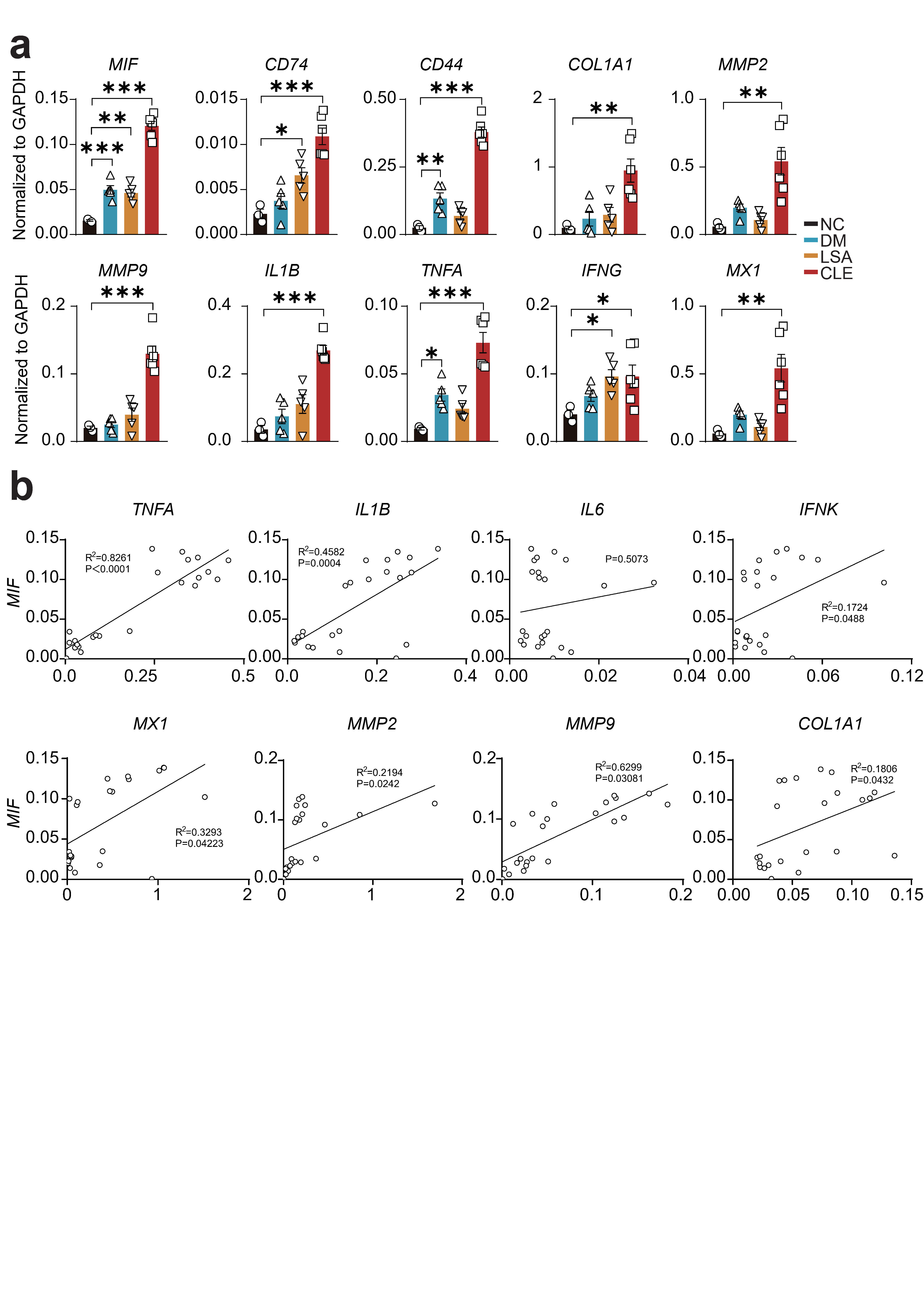

Supplement: Supplementary file 3 — Fig. S2 [file 41419_2026_8443_MOESM3_ESM.jpg]

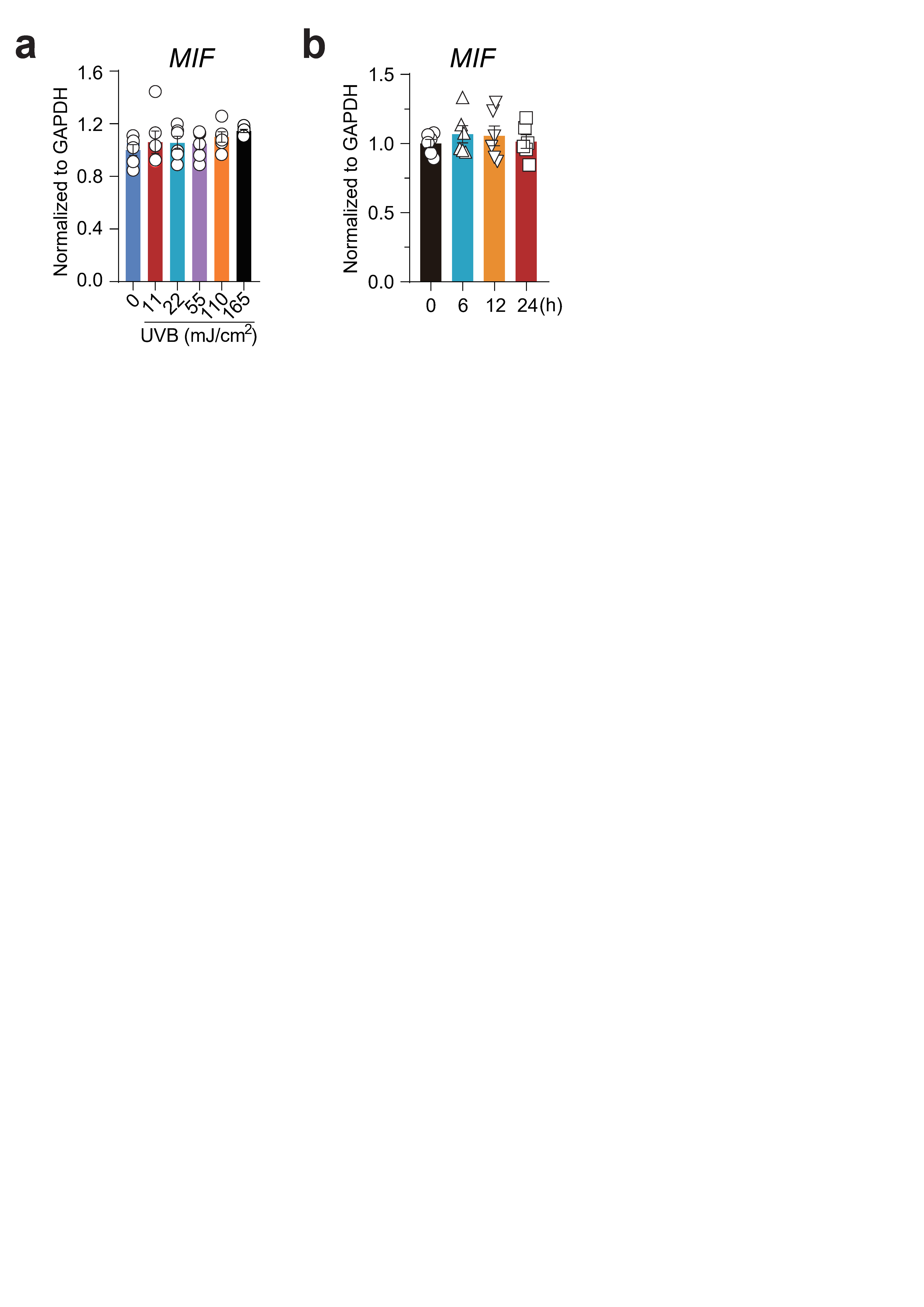

Supplement: Supplementary file 4 — Fig. S3 [file 41419_2026_8443_MOESM4_ESM.jpg]

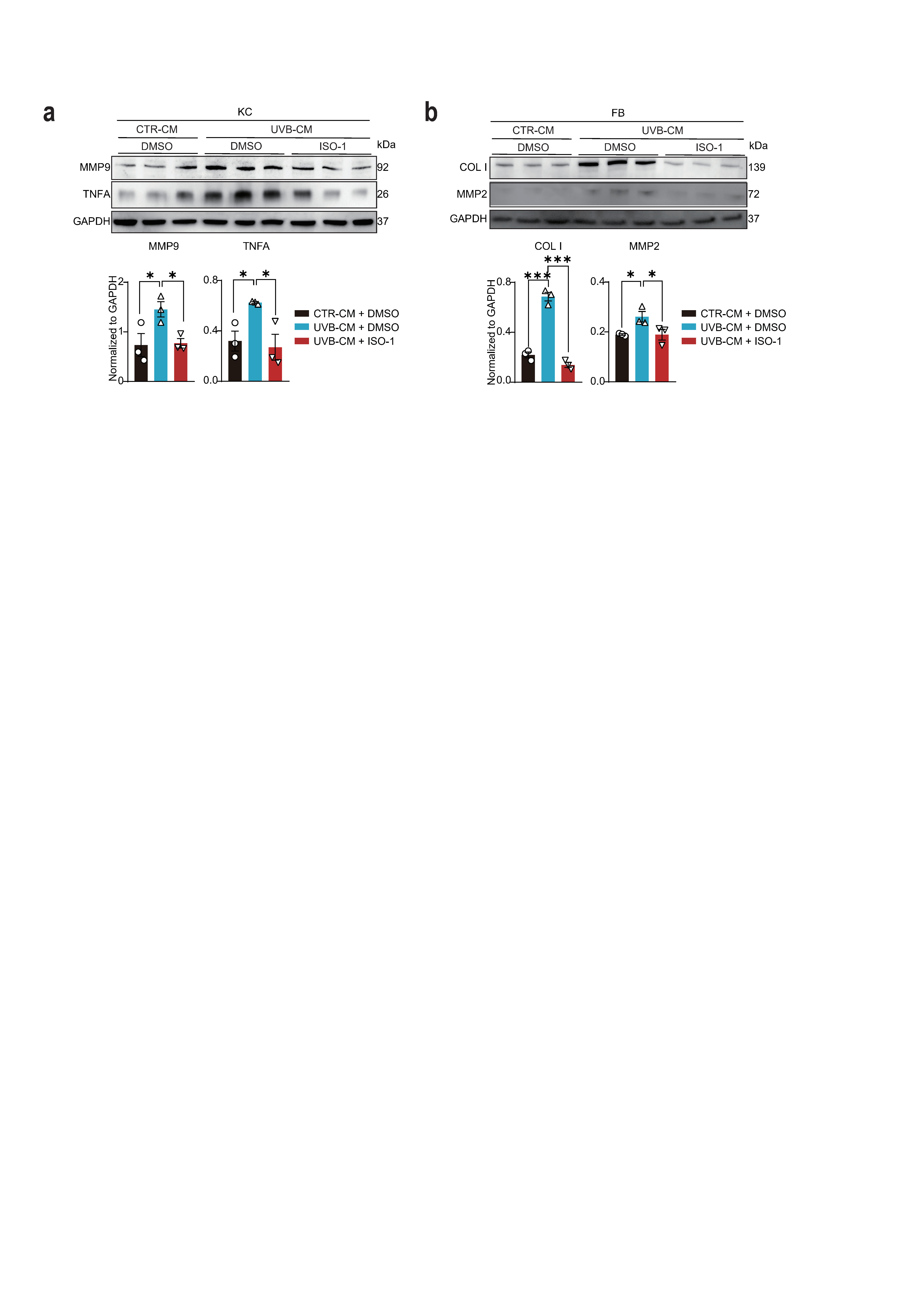

Supplement: Supplementary file 5 — Fig. S4 [file 41419_2026_8443_MOESM5_ESM.jpg]

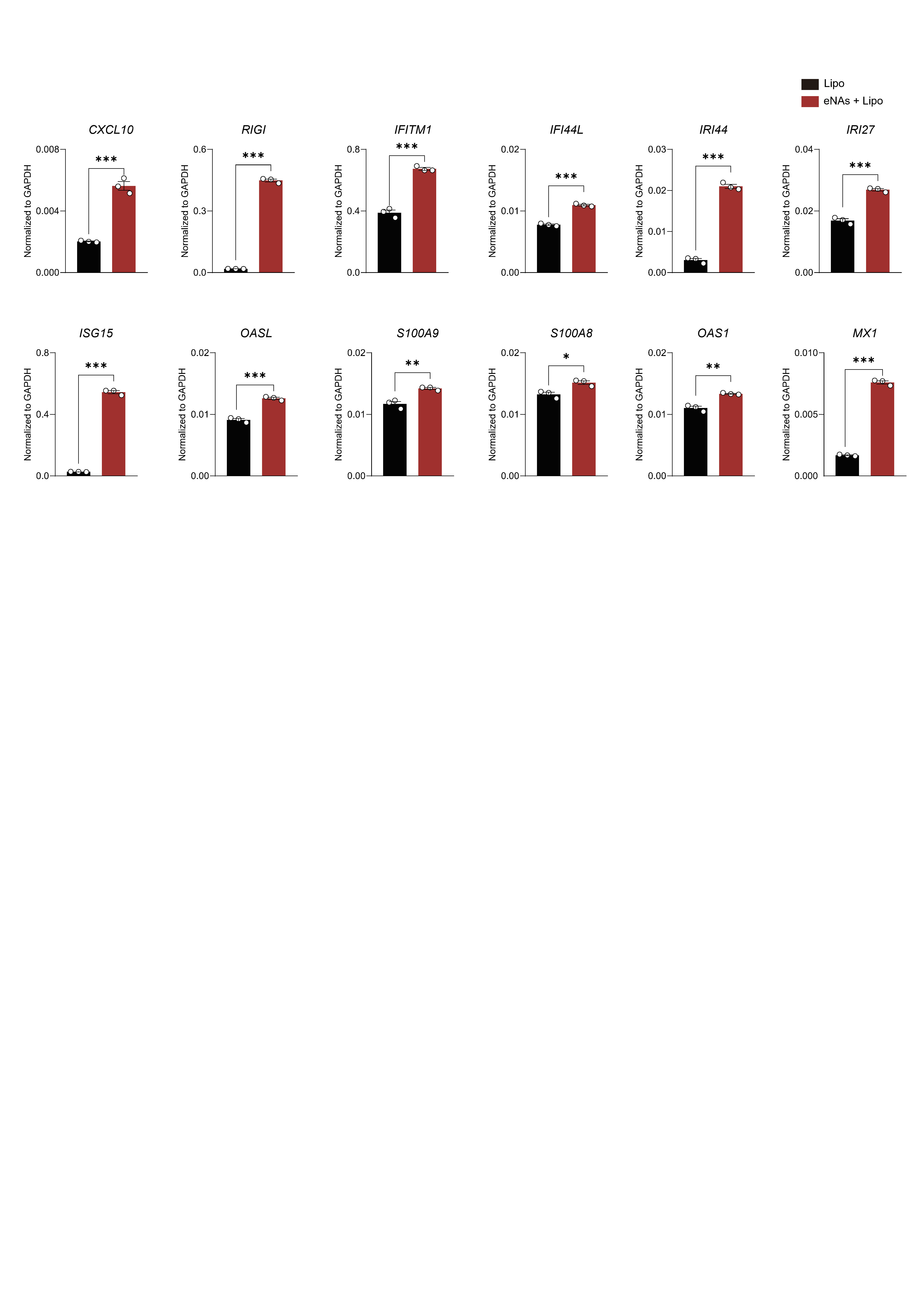

Supplement: Supplementary file 6 — Fig. S5 [file 41419_2026_8443_MOESM6_ESM.jpg]

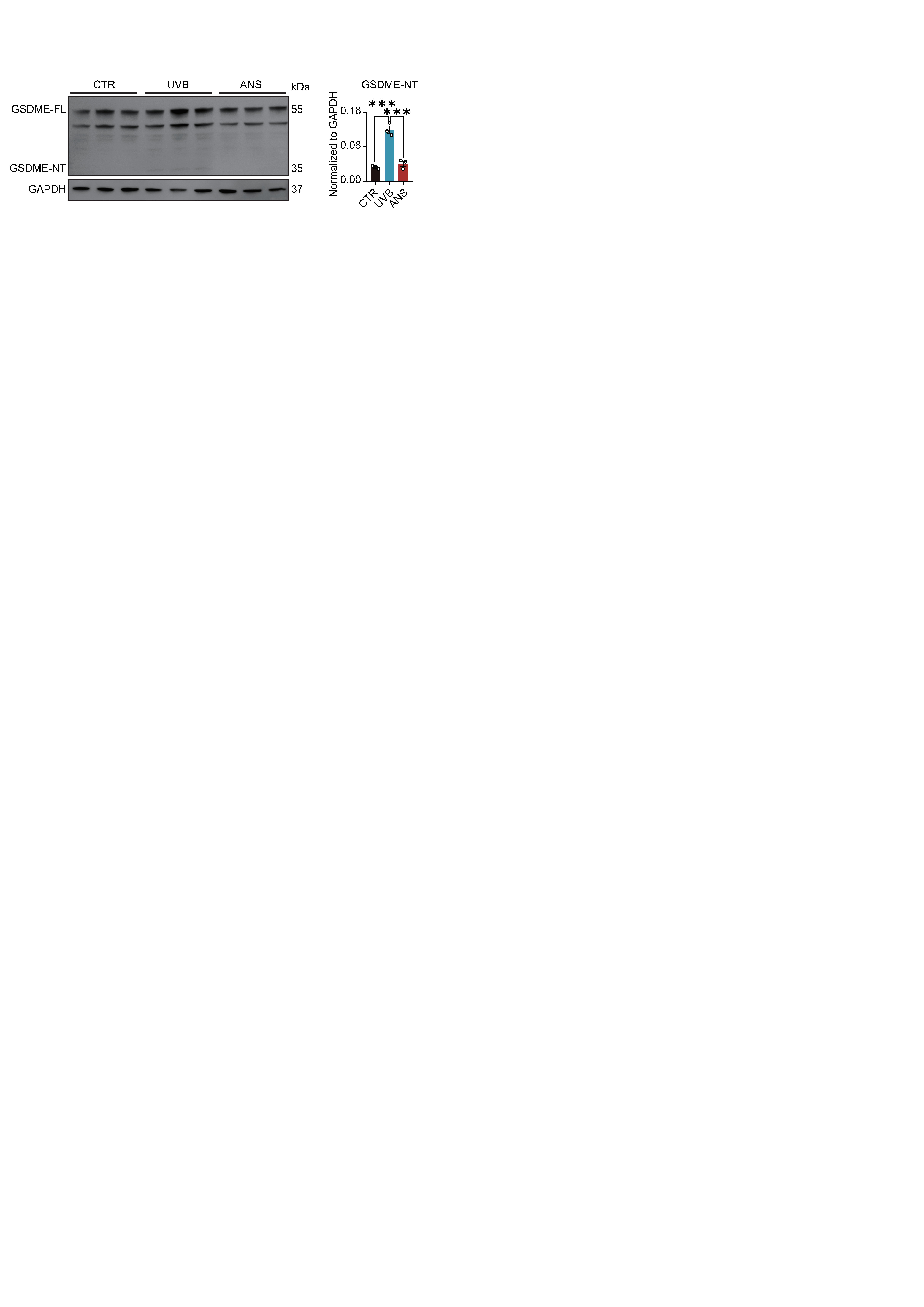

Supplement: Supplementary file 7 — Fig. S6 [file 41419_2026_8443_MOESM7_ESM.jpg]

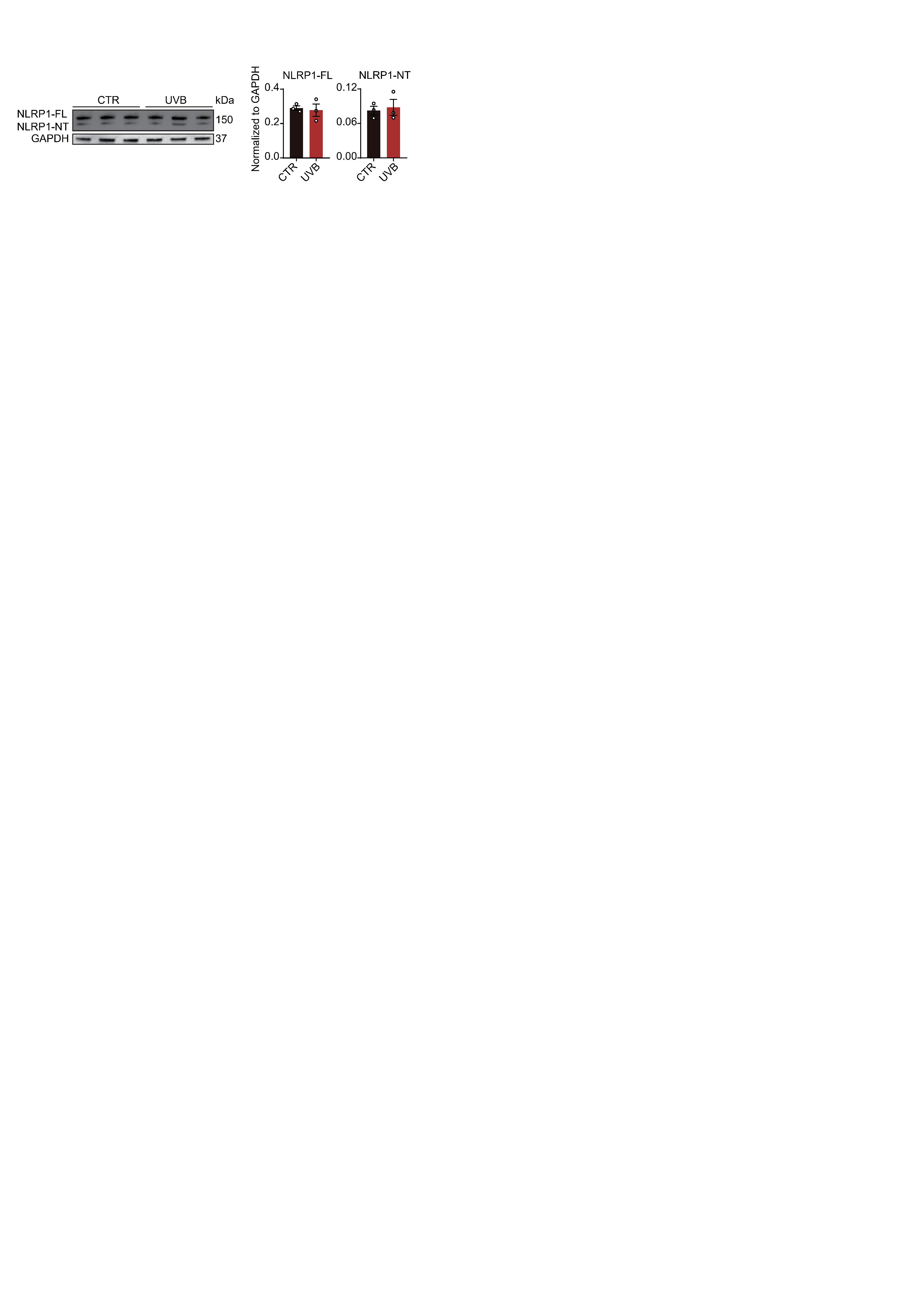

Supplement: Supplementary file 8 — Fig. S7 [file 41419_2026_8443_MOESM8_ESM.jpg]

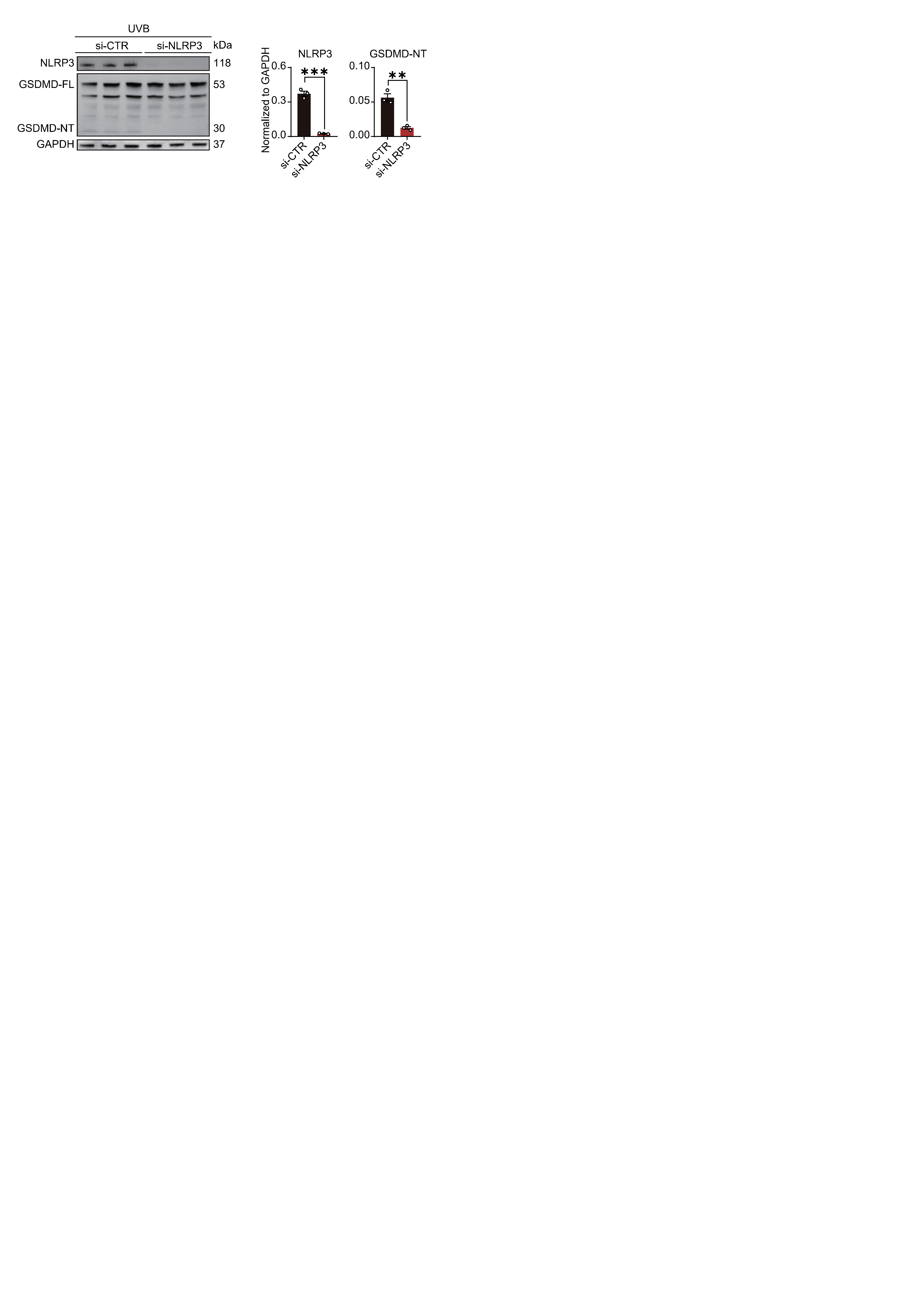

Supplement: Supplementary file 9 — Fig. S8 [file 41419_2026_8443_MOESM9_ESM.jpg]

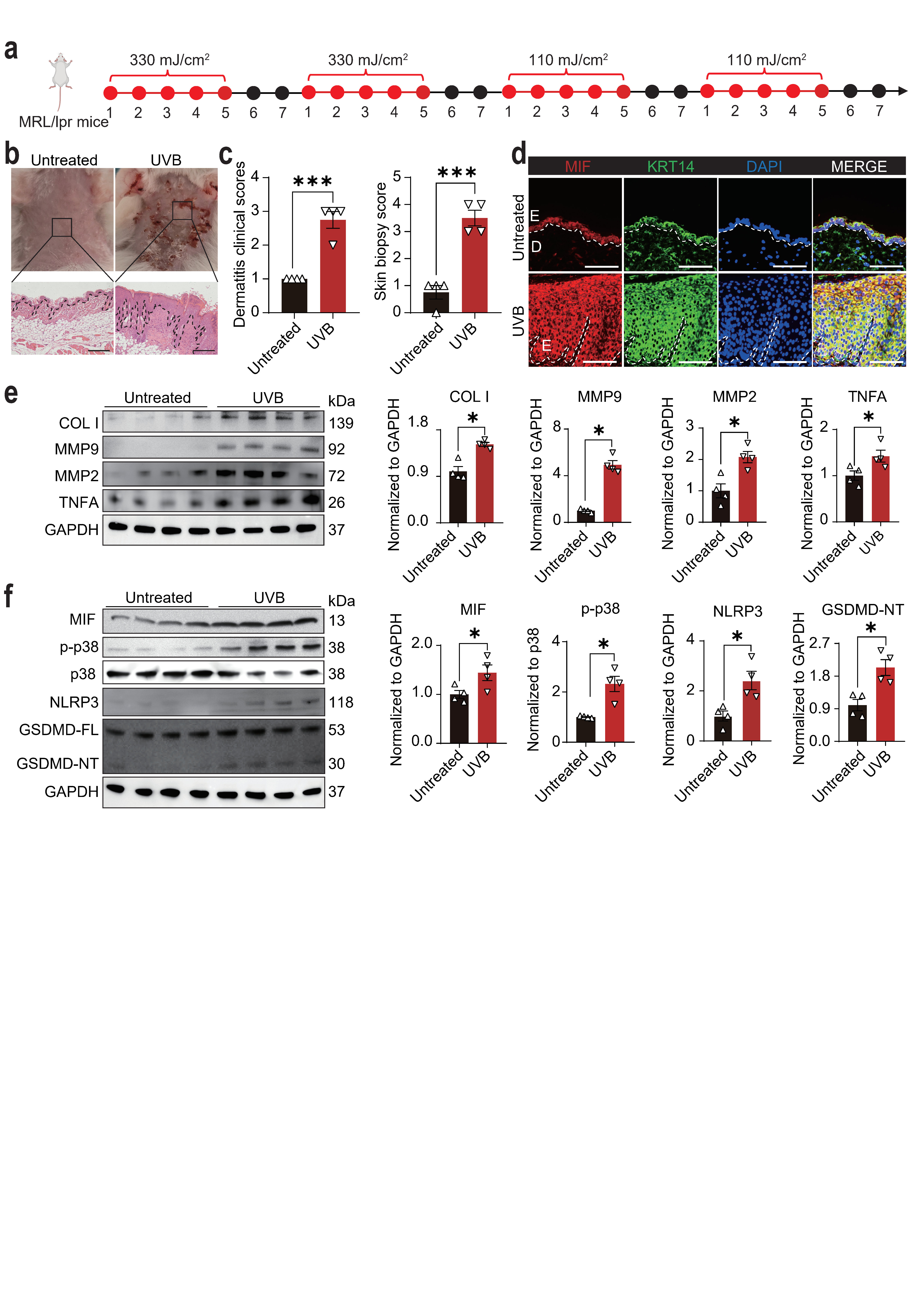

Supplement: Supplementary file 10 — Fig. S9 [file 41419_2026_8443_MOESM10_ESM.jpg]

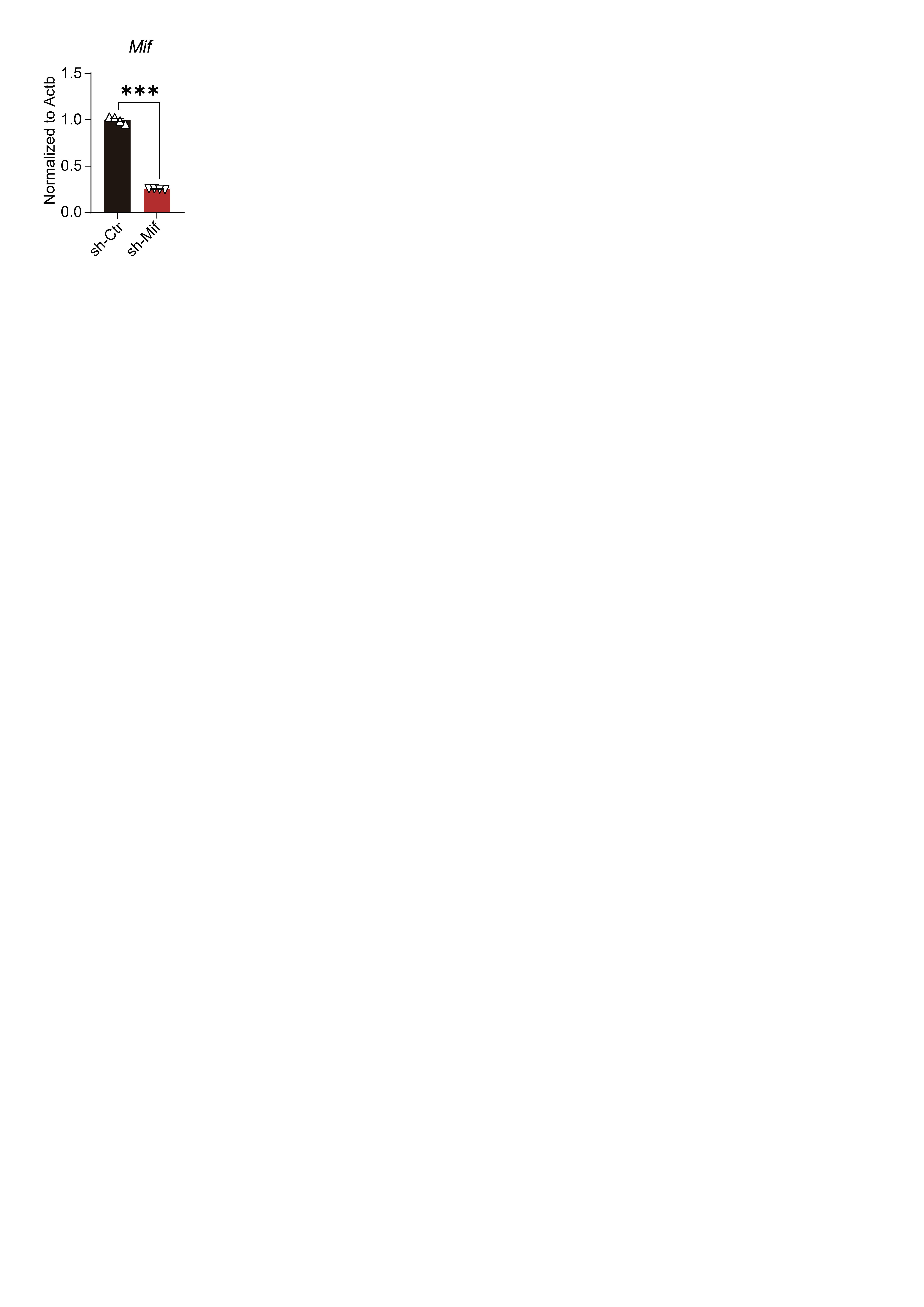

Supplement: Supplementary file 11 — Fig. S10 [file 41419_2026_8443_MOESM11_ESM.jpg]

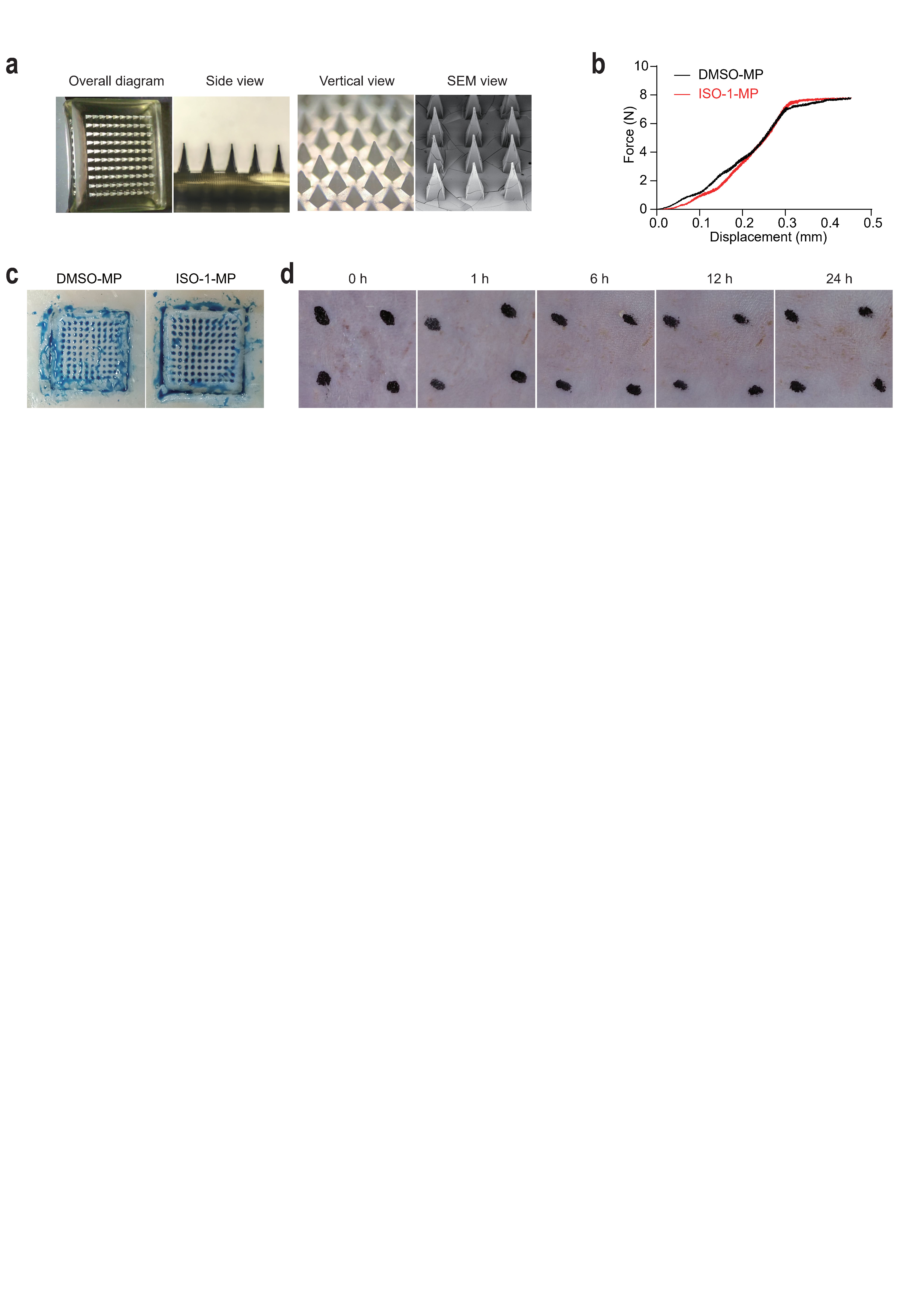

Supplement: Supplementary file 12 — Fig. S11 [file 41419_2026_8443_MOESM12_ESM.jpg]

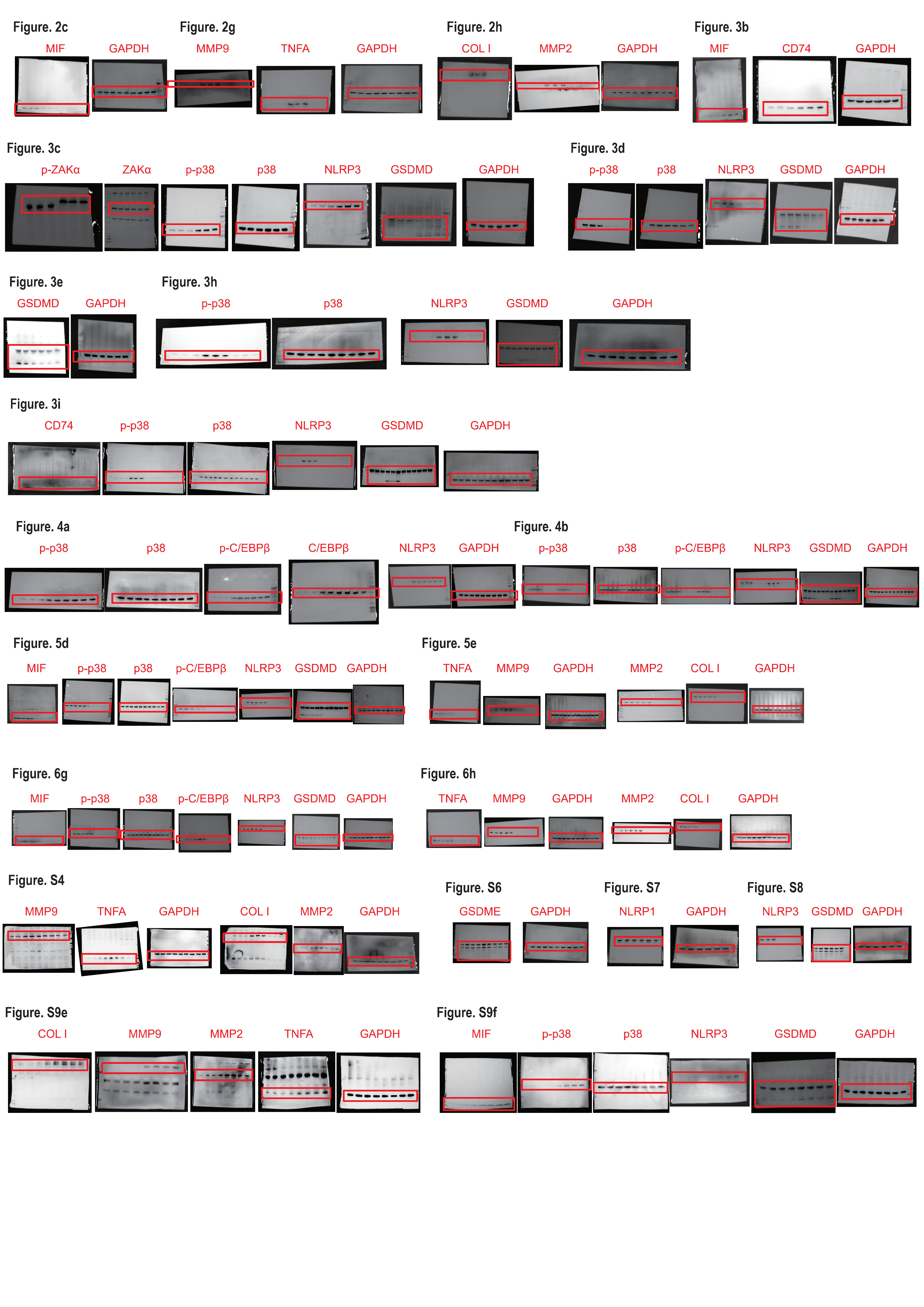

Supplement: Supplementary file 13 — Fig. S12 [file 41419_2026_8443_MOESM13_ESM.jpg]
